# Unpacking weight management interventions measuring eating disorder risk in adults: coding of components of interventions in a systematic review

**DOI:** 10.1186/s40337-026-01667-x

**Published:** 2026-06-17

**Authors:** Natalie B. Lister, Rabia Khalid, Isabelle R. Jardine, Samantha Pryde, Hannah Melville, Anna L. Seidler, Kylie E. Hunter, Amy L. Ahern, Louise A. Baur, Caroline Braet, Sarah P. Garnett, Andrew J. Hill, Sarah Maguire, Dasha Nicholls, Susan J. Paxton, Milan K. Piya, Amanda Sainsbury, Katharine Steinbeck, Denise E. Wilfley, Kelly Cooper, Genevieve Dammery, Alicia M. Grunseit, Faith Anne N. Heeren, Rebecca A. Jones, Theodore K. Kyle, Fiona Quigley, Molly Robbins, Aviva H. Ariel-Donges, Rachel D. Barnes, Kristine Beaulieu, Kerri N. Boutelle, Simona Calugi, Kelly M. Carpenter, Hoi Lun Cheng, Riccardo Dalle Grave, Marcelo Demarzo, Dawn M. Eichen, Erin L. Glynn, Katrijn Houben, Corby K. Martin, Janell L. Mensinger, Jayanthi Raman, Kristin M. von Ranson, Hollie A. Raynor, Elizabeth Rieger, Eric Robinson, Vera Salvo, Nancy E. Sherwood, Sharon A. Simpson, Evelyn Smith, Rachael W. Taylor, Krista A. Varady, Victoria Whitelock, Brittany J. Johnson, Hiba Jebeile

**Affiliations:** 1 The Children’s Hospital Westmead Clinical School, The University of Sydney, Sydney, Australia; 2 Charles Perkins Centre, The University of Sydney, Sydney, Australia; 3 Caring Futures Institute, College of Nursing and Health Sciences, Flinders University, Adelaide, Australia; 4 National Health and Medical Research Council Clinical Trials Centre, The University of Sydney, Sydney, Australia; 5 Department of Child and Adolescent Psychiatry, University Medical Centre Rostock, Rostock, Germany; 6 Partner Site Greifswald/Rostock, German Center for Child and Adolescent Health (DZKJ), Rostock, Germany; 7 MRC Epidemiology Unit, University of Cambridge, Cambridge, UK; 8 Weight Management Services, Children’s Hospital at Westmead, Sydney, Australia; 9 Department of Developmental, Personality and Social Psychology, Ghent University, Ghent, Belgium; 10 Kids Research, Children’s Hospital at Westmead, Sydney, Australia; 11 Leeds Institute of Health Sciences, University of Leeds, Leeds, UK; 12 InsideOut Institute for Eating Disorders, Boden Collaboration for Obesity, Nutrition and Eating Disorders, Charles Perkins Centre, The University of Sydney, Sydney, Australia; 13 Division of Psychiatry, Imperial College London, London, UK; 14 Applied Research Collaboration, National Institute for Health and Care Research, London, UK; 15 School of Psychology and Public Health, La Trobe University, Melbourne, Australia; 16 School of Medicine, Western Sydney University, Campbelltown, Australia; 17 Camden and Campbelltown Hospitals, Campbelltown, Australia; 18 School of Human Sciences, University of Western Australia, Perth, Australia; 19 Lifetime Associate, Childrens Hospital at Westmead, Sydney, Australia; 20 Faculty of Medicine and Health, The University of Sydney, Sydney, Australia; 21 School of Medicine, Washington University in St. Louis, St Louis, USA; 22 Weight Issues Network, Sydney, Australia; 23 Department of Nutrition and Dietetics, Childrens Hospital at Westmead, Sydney, Australia; 24 Department of Implementation Science, School of Medicine, Wake Forest University, Winston-Salem, USA; 25 WW International, New York, USA; 26 ConscienHealth, Pittsburgh, USA; 27 Institute of Nursing and Health Research, School of Communication and Media, Ulster University, Belfast, UK; 28 College of Psychology, Nova Southeastern University, Fort Lauderdale, USA; 29 Reciprocity Health, Wilmington, USA; 30 Medical School, University of Minnesota, Minneapolis, USA; 31 School of Psychology, Faculty of Medicine and Health, University of Leeds, Leeds, UK; 32 Steno Diabetes Center Copenhagen, Copenhagen University Hospital, Copenhagen, Denmark; 33 Department of Pediatrics, University of California San Diego, San Diego, USA; 34 Department of Eating and Weight Disorders, Villa Garda Hospital, Verona, Italy; 35 Center for Wellbeing Research, RVO Health, Seattle, USA; 36 The Academic Department of Adolescent Medicine, Children’s Hospital at Westmead, Sydney, Australia; 37 Mente Aberta—The Brazilian Center for Mindfulness and Health Promotion, Federal University of São Paulo, São Paulo, Brazil; 38 Nuchi Health LLC, Salt Lake City, USA; 39 Department of Clinical Psychological Science, Faculty of Psychology and Neuroscience, Maastricht University, Maastricht, Netherlands; 40 Ingestive Behavior, Weight Management & Health Promotion Laboratory, Pennington Biomedical Research Center, Baton Rouge, USA; 41 Department of Clinical and School Psychology, Nova Southeastern University, Fort Lauderdale, USA; 42 School of Psychological Sciences, University of Newcastle, Callaghan, Australia; 43 Department of Psychology, University of Calgary, Calgary, Canada; 44 Department of Nutrition, University of Tennessee, Knoxville, USA; 45 School of Medicine and Psychology, Australian National University, Canberra, Australia; 46 Department of Psychology, University of Liverpool, Liverpool, UK; 47 Division of Epidemiology and Community Health, School of Public Health, University of Minnesota, Minneapolis, USA; 48 Medical Research Council/Chief Scientist Office, Social and Public Health Sciences Unit, School of Health and Wellbeing, University of Glasgow, Glasgow, UK; 49 School of Psychology, Western Sydney University, Sydney, Australia; 50 Department of Medicine, University of Otago, Dunedin, New Zealand; 51 Department of Kinesiology and Nutrition, University of Illinois at Chicago, Chicago, USA; 52 Evidence and Implementation Department, Cancer Research UK, London, UK

**Keywords:** Obesity, Overweight, Disordered eating, Behavior change techniques, Obesity treatment

## Abstract

**Introduction:**

Adult weight management interventions are complex; better understanding of the intervention components that may impact eating disorder (ED) risk is required.

**Methods:**

Weight management randomized controlled trials (RCTs) for adults with overweight/obesity that measured ED risk were systematically searched in four databases and two trial registries. A project-specific codebook was used to code 84 delivery features and 89 intervention strategies of trials. Individual strategies were grouped into 20 clusters which were further grouped into five broad categories. Trial investigators verified coding and narrative synthesis using descriptive statistics of findings was reported.

**Results:**

Of 14,880 identified, 58 eligible trials were coded, of which 26 trials with 64 intervention arms were verified and therefore included. Intervention arms included a mean (SD) of 24 (11) intervention strategies. Commonly used intervention strategy clusters were nutrition education (91%), dietary behavior change strategies (84%), physical activity education (81%), and dietary self-monitoring (80%). Few interventions used strategies in the category of psychological components (13–41%). The median (range) intervention duration was 27 (4–104) weeks, and contacts with participants typically included a staged approach of weekly to monthly contact.

**Conclusion:**

Adult weight management interventions are multifactorial with varying delivery features and intervention strategies. Despite this, psychological (e.g. weight stigma) and sleep-health related strategies are either rarely used or are underreported. Breaking down intervention components using our framework can help identify which strategies influence outcomes, including eating disorder risk, and inform the design and reporting of future interventions.

**Plain English summary:**

Some adults seeking weight management for obesity may have an eating disorder or disordered eating behaviors. Research shows most people who take part in behavioral weight management programs have an improvement in eating disorder symptoms, however a small number may experience worsening symptoms. In this study, we break down behavioral weight management programs that measure eating disorder risk to better understand the features they use. We found that these programs vary widely in the number and type of strategies they use, as well as in how they are delivered, such as the length of the program. Strategies that focus on psychological factors (for example, addressing weight stigma) and sleep health were rarely used or reported. These findings are useful in guiding the design and reporting of future behavioral weight management programs. They can also be used in future research to determine which specific program features improve or worsen outcomes such as eating disorder risk.

**Supplementary Information:**

The online version contains supplementary material available at 10.1186/s40337-026-01667-x.

## Introduction

In adults seeking behavioral weight management for obesity, a proportion have eating disorders or disordered eating behaviors [1]. Behavioral weight management usually includes dietary, physical activity and psychological strategies to promote weight loss and improve health. It is first line treatment for obesity and foundational to adjunctive therapies (e.g., obesity medications, metabolic and bariatric surgery [2–5]. Some behavioral weight management interventions have been shown to improve both obesity and disordered eating outcomes [6, 7], likely because they incorporate components similar to those used in eating disorder treatment [8]. While recommendations exist on approaches to behavioral weight management [9], interventions often vary in duration and dose, the strategies used to change behaviors (e.g., dietary monitoring and goals setting) and the way they are delivered (e.g. group vs individual). Traditional evidence synthesis typically does not examine how variations in these intervention components may influence outcomes. Both intervention effectiveness and safety may be influenced, and this has important implications for evidence translation.

The Eating Disorders in weight-related Therapies (EDIT) Collaboration [10] consists of international researchers, clinicians and people with lived experience, and brings together randomized controlled trials (RCTs) to identify risk factors and components of behavioral weight management interventions that may contribute to eating disorder risk. For example, dietary restraint, or consciously trying to limit food intake to control body weight, which is common in weight management interventions, has been suggested to increase eating disorder risk in observational studies [11]. However in pediatric populations, dietary restraint may not be associated with eating disorder risk within the context of weight management [12]. Components such as dietary or weight monitoring have also been suggested to increase disordered eating behaviors [13], whereas studies of long-term weight maintenance suggest self-monitoring improves outcomes [14]. Other intervention features, such as addressing sleep health and body image concerns may also decrease risk [15]. Detailing intervention components used in weight management will improve our understanding of such interventions. Importantly, this will help future studies to determine which components, or combination of intervention components, may influence a person’s risk to eating disorders when undertaking behavioral weight management. To examine components of weight management interventions in the context of eating disorder risk, our team developed a project specific framework of intervention components based on broad stakeholder consultation [15, 16]. The framework consists of *intervention strategies*, which include behavior change techniques (BCTs) and content specific to weight management interventions and *delivery features*, referring to how the interventions were delivered [17]. When applying this framework to adolescent weight management trials [18], we found that interventions are multidimensional and vary widely in delivery features and intervention strategies. Given that weight management for adolescents and adults differs [19], with each described separately in clinical guidelines [4, 5], a separate examination of adult interventions is warranted.

Breaking down and understanding behavioral weight management interventions is an essential first step to filling a gap in research and designing effective weight management interventions that are also protective against eating disorders. A recent clinical advisory on GLP-1 therapy for obesity reinforces this through emphasizing the importance of nutrition and lifestyle interventions alongside treatment, and prioritizing management of disordered eating [20]. This review aimed to describe the delivery features and intervention strategies of behavioral weight management interventions that measure eating disorder outcomes in adults.

## Methods

This systematic review was registered on the PROSPERO International prospective register of systematic reviews (CRD42021265340). The protocol detailing the intervention coding design was published on Open Science Framework. Minor modifications were made to the protocol following registration and are listed in Supplementary file 1 [21]. This review contributes to a series of complementary projects conducted by the EDIT Collaboration [10, 18, 21]. Project methods were adapted from those used by the Transforming Obesity Prevention for CHILDren (TOPCHILD) Collaboration [22]; detailed project descriptions have been published elsewhere [10, 16, 21], and methods specific to this review are described below. Reporting followed the Preferred Reporting Items for Systematic review and Meta-Analysis (PRISMA) checklist [23].

### Eligibility criteria

Eligible studies included randomized controlled trials recruiting adults (≥ 19 years) with overweight or obesity, as reported by the trial or defined as, body mass index [(BMI) ≥ 25 kg/m^2^]. A separate analysis has been conducted for adolescents which is published elsewhere [18]. Trials were required to meet the following criteria: (1) include at least one behavioral weight management intervention aiming to improve a weight-related outcome (e.g., weight maintenance or weight loss); (2) have a comparison group (e.g., wait-list/no-treatment control, standard care, alternate intervention); and (3) assess eating disorder symptoms at baseline and post-intervention or follow-up using a validated assessment tool (e.g. Eating Disorder Examination Questionnaire, Binge Eating Scale). Trials were excluded if interventions included bariatric surgery, post-surgical interventions, obesity pharmacotherapy, nutrient supplementation, or those targeting secondary or syndromic causes of obesity, alternate medical conditions, eating disorders, or obesity prevention.

### Information sources, search strategy and selection process

Studies were identified through systematic searches of four electronic databases (MEDLINE, Embase, PsycINFO and Scopus) from inception to March 2022, and clinical trial registries [ClinicalTrials.gov and the World Health Organization’s International Clinical Trials Registry Platform Search Portal [24]] in May 2022. The search strategy for each database is outlined in Supplementary file 2. Two independent reviewers used Covidence online software (Veritas Health Innovation Ltd, Australia) to screen records by title/abstract and then full text. Disagreements were resolved through discussion or a third reviewer. Additional eligible studies were identified through professional networks and hand searching.

### Data collection

Study characteristics, including trial name, location, year(s) of recruitment, participant characteristics, and number of intervention arms, were extracted by a single reviewer and checked by a second reviewer. The primary data sources were published protocols and manuscripts. If a secondary outcome paper was identified in the systematic search, the protocol and primary outcome papers were sourced for coding purposes.

### Coding framework

A project specific intervention component framework of delivery features and intervention strategies was developed and piloted, with details published elsewhere [16]. In short, the research team identified possible components of weight management interventions that may influence eating disorder risk through consultation with stakeholders, including clinicians, researchers, and individuals with lived experience of eating disorders and/or obesity [15]. These components were combined in a detailed codebook [16]. Each unique code in the codebook referred to an individual delivery feature or intervention strategy with a detailed definition (Supplementary file 3). Following the publication of the protocol, one new intervention strategy was added: ‘Language’: weight-focused or health-focused language, or a combination [21].

Delivery features refer to “intervention characteristics that relate to how an intervention is delivered” [25]. These were adapted from the Template for Intervention Description and Replication (TIDieR) checklist [17], and included the ‘Why’ (overarching goal, psychological theory or framework); ‘Target population/recipient of the intervention’ (age group, weight category, support); ‘What’ (materials provided, procedures used, outcome measures); ‘Who’ (delivered the intervention and training for interventionist); ‘How’ (delivery mode, individual/group); ‘Where’ (intervention setting); ‘When and how much’ (intervention dose and post-intervention support); tailoring; modifications; and ‘How well’ (fidelity). When examining whether psychological or mental health outcomes were measured by trials, this did not include eating disorder outcomes, as all eligible trials were required to assess this.

Intervention strategies describe the behavior change content of weight management interventions with consideration of eating disorder risk. The 89 distinct intervention strategies (i.e., lowest-level grouping), were categorized into 20 clusters of similar strategies (i.e., mid-level grouping), which were further grouped into five categories (i.e., highest-level grouping). Figures in supplementary file 3 portray these five categories, which include: intervention intent, framing and outcomes (2 clusters; 13 strategies); dietary strategies (5 clusters; 27 strategies); eating behaviors/disordered eating (2 clusters; 10 strategies); movement and sleep related strategies (6 clusters; 23 strategies); and psychological health related strategies (5 clusters; 16 strategies). All delivery features and intervention strategies are based on previously tested ontologies [25, 26] and were refined through stakeholder consultation and pilot testing [16, 25].

### Intervention coding procedures

Intervention components (delivery features and intervention strategies) were coded using the study-specific framework. Coding was completed for all active intervention arms, (i.e. those that provided any intervention including control arms with usual care). Waitlist or no treatment control arms were not considered active interventions and were not coded. For each intervention, individual components were coded as present, absent, or maybe present. Coding was undertaken by an accredited practicing dietitian (RK) and a researcher with a psychology background (SP), who were trained in using the study-specific coding manual. Coding procedures were refined during the piloting of the framework [16]. Each trial was coded independently in duplicate by both coders until an acceptable level of interrater agreement in coding (75%) was reached [27, 28], after which coding was completed by a single coder. Regular meetings were held within the coding team to resolve any uncertainties. All intervention components were coded by the study team, with the exception of “Language” in the cluster of ‘Framing of the intervention (communication strategies)’. This component referred to language used with participants and was reported by trialists during verification of codes as described below.

Trial investigators, referred to as trialists hereon, were invited to validate coding as part of a virtual meeting or by email. Verification has been shown to increase the accuracy of intervention coding [29]. This involved confirming codes and removing all “maybe” codes. If trialists were uncertain about any “maybe” codes, these were recoded as “no”. Conflicts in coding reported by trialists were resolved by the coding team through discussion. Percentage agreement between our coding and trialist-verified coding was calculated as the number of matching codes divided by the total number of coding comparisons, multiplied by 100. All coding data were entered into a REDCap database (Research Electronic Data Capture tools, hosted by the University of Sydney) [30, 31].

### Synthesis methods

As trials commonly included more than one active intervention, the intervention arms were the unit of analysis. Descriptive statistics were used to identify the frequency (count and percent) of each component across all intervention arms. Analyses were completed in SPSS Statistics, version 28.0 (IBM Inc). For each intervention arm, the proportion of strategies used was displayed using heat maps. Flourish (Kiln Enterprises Ltd. http://flourish.studio) was used to develop network maps to illustrate the frequency of, and relationships between intervention strategy clusters across intervention arms. Due to changes in coding following verification with trialists, only validated intervention arms were included in the primary analyses. Sensitivity analyses were undertaken with all eligible trials, using coded data based on published materials only (i.e. coded data without updates from the verification procedures).

## Results

### Study selection and sample characteristics

**Fig. 1:**
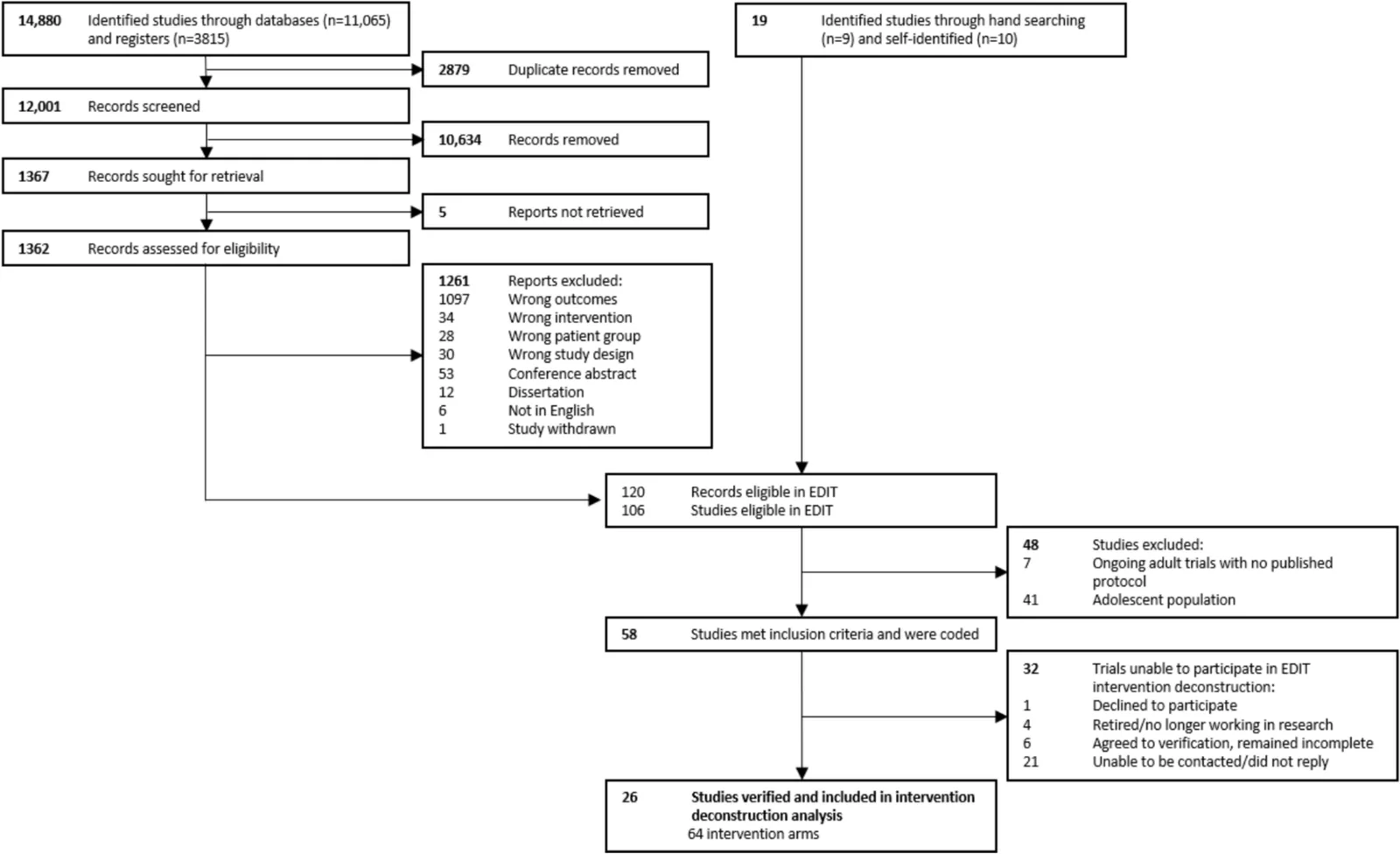
PRISMA flow diagram of study selection. Records eligible for EDIT included all relevant publications, which included multiple secondary papers of trials, whereas studies eligible for EDIT refers to individual trials

Of the 14,880 articles identified in the search, 58 trials were eligible and coded [32–116]. Twenty-six trials [75–116] with 64 intervention arms (k) had their coding verified with trialists and were included in the primary analysis (Fig. 1). Trials were published between 2006 and 2023 and were most frequently conducted in the United States (11 out of 26), Australia (6 out of 26) and the United Kingdom (3 out of 26). See Table 1 for the description of study characteristics.

**Table 1:** Characteristics of included studies

Study details [First author, Year of publication(s); Country; Setting (hospital outpatient/inpatient, university, community etc.)]	Participant characteristics*: [Sample size (n), %female (F), mean age (SD) in years (y)]	Intervention details: [Intervention duration, Duration of follow-up (FU) (from post-intervention); Delivery, intensity (individual or group setting, frequency of contact)]
Barnes et al. 2014 [81]; Barnes et al. 2017 [82]; USA; Primary care & virtual	n = 89 76.4% (F) *MIC* 47.07 (9.97) y, *NPC* 48.98 (11.59) y, *UC* 47.77 (10.05) y	*MIC* & *NPC*: 3 months, FU 3- and 12-months post-intervention Individual, every 3 weeks
Beaulieu et al. 2019 [83]; United Kingdom; University	n = 46 100% (F), *CER* 34 (9) y, *IER* 35 (11) y	< 12 weeks, no FU Individual, weekly
Boutelle et al. 2022 [87]; Boutelle et al. 2019 [88]; USA; University	n = 271 81.6% (F), 46.97 (11.8) y	1 year, FU 12 months post-intervention Group, staged approach (e.g. weekly > monthly)
Carpenter et al. 2019 [84]; USA; Virtual	n = 75 92% (F), 47.3 (10.0) y	6 months, no FU Individual, weekly
Cheng et al. 2014 [85]; Griffin et al. 2013 [86]; Australia; Hospital outpatient	n = 71 100% (F), *HP* 22.4 (0.5) y, *HC* 22.1 (0.5) y	12 months, no FU Individual, staged approach (e.g., weekly > monthly)
Dalle Grave et al. 2013 [117]; Italy; Hospital inpatient & outpatient	n = 88 58% (F), 46.7 (11.1) y	1 year, no FU Group, staged approach (e.g., weekly > monthly)
Dassen et al. 2018 [90]; The Netherlands; Virtual	n = 91 74.7% (F), 47.97 (10.69) y	< 8 weeks, FU 6 months post-intervention Individual, less than weekly
Glynn et al. 2022 [111]; Canada & USA; Community	n = 206 70% (F), *LPF* 36.1 (7.7) y, *HPF* 37.9 (7.9) y	12 weeks, no FU Individual, greater than monthly
Jospe et al. 2017 [91]; Taylor 2015 [92]; New Zealand; University	n = 250 *Control* 46.7 (11.4) y, 62.5% (F) *Daily weighing* 46.1 (11.4) y, 62.8% (F) *MyFitnessPal* 44.4 (10.2) y, 62% (F) *Brief support* 40.6 (9.9) y, 60.8% (F) *Hunger training* 40.7 (10.8) y, 62% (F) *Overall mean age not provided*	12 months, no FU All arms used same delivery, i.e. individual, but differed in intensity *Control:* less than weekly *Daily weighing:* less than weekly *MyFitnessPal:* staged approach (e.g. weekly > monthly) *Brief support:* monthly *Hunger training:* staged approach (e.g. weekly > monthly)
Lin et al. 2023 [112]; USA; University	n = 90 82.2% (F), *TRE* 44 (12) y, *Daily CR* 44 (9) y, *Control* 44 (13) y	*TRE* & *Daily:* 12 months, no FU Individual, staged approach (e.g. weekly > monthly)
Martin et al. 2019 [105]; Myers et al. 2014 [106]; USA; University	n = 171 72.5% (F), 48.9 (11.4) y	6 months, no FU All arms used same delivery, i.e. individual, but differed in intensity *8KKW* & *20KKW*: less than weekly *Control*: staged approach (e.g., weekly > monthly)
Mensinger et al. 2016 [118]; Mensinger et al. 2016 [96]; USA; Hospital outpatient	n = 80 100% (F), *Weight-neutral program* 39.83 (4.34) y, *Weight-loss* 39.35 (3.91) y	6 months, FU 18 months post-intervention Group, weekly
Moss et al. 2017 [119]; Canada; University	n = 135 77.8% (F), 45.16 (11.30) y	12 weeks, FU 6 months post-intervention Group, weekly
Perri et al. 2014 [115]; Ariel & Perri 2014; USA; Community	n = 612 78.3% (F), 52.3 (11.5) y	2 years, no FU Group, staged approach (e.g. weekly > monthly)
Raman et al. 2018 [97]; Australia; University	n = 80 86% (F), *Control* 42.2 (8.8) y, *Treatment* 40.6 (7.0) y	7–9 weeks, FU 3 months post-intervention Group, weekly
Rieger et al. 2017 [100]; Rieger et al. 2013 [101]; Australia; University & Community	n = 201 73.6% (F), *CBT-A* 46.93 (12.01) y, *CBT-SP* 47.1 (11.0) y	1 year, FU 1 year post-intervention Group, staged approach (e.g. weekly > monthly)
Raynor et al. 2006 [99]; USA; University	n = 30 90% (F), 49.5 (9.5) y	8 weeks, FU 1 week post-intervention Group, weekly
Raynor et al. 2012 [94]; LaRose 2014 [95]; USA; University	n = 202 57.8% (F), 51.3 (9.5) y	18 months, no FU Group, staged approach (e.g., weekly > monthly)
Salvo et al. 2022 [120]; Salvo et al. 2018 [121]; Brazil; Primary care	n = 284 100% (F), 40.4 (10.7) y	10 weeks, FU 3 months post-intervention Group, weekly
Seimon et al. 2019 [75]; Seimon et al. 2018 [76]; Australia; University & virtual	n = 101 100% (F), 58.0 (4.2) y	12 months, FU 2 years post-intervention Individual, monthly
Sherwood et al. 2011 [107]; Pacanowski et al. 2014; USA; Virtual (phone)	n = 419 81.6% (F), 47.0 (10.8) y	Each arm had a different intervention duration, FU period and differed in intensity *Guided maintenance:* 24 months, no FU Individual, staged approach (e.g. weekly > monthly) *Self-directed maintenance:* 1 month, FU 23 months post-intervention Individual, every 2–3 weeks
Simpson et al. 2015 [102]; United Kingdom; Community	n = 170^a^ Data for n = 166: 84.3% (F), < 30 y 9.6% (F), 30–59 y 60.8% (F), ≥ 60 29.5% (F) *Overall mean age not provided*	12 months, no FU Both arms used same delivery, i.e. individual, but differed in intensity *High* & *low contact:* staged approach (e.g. weekly > monthly) *Control:* intensity could not be calculated
Smith & Whittingham 2017 [103]; Australia; Community	n = 176 *Ongoing study with no published data. Inclusion criteria: 18-55y*	20 weeks, FU 12 months post-intervention Group, weekly
Williamson et al. 2008 [78]; Anton et al. 2008 [79]; Heilbronn et al. 2006 [80]; USA; University	n = 48 56% (F), *Control* 37 (2.1) y, *CR* 39 (1.5) y, *CR* + *EX* 36 (1.6) y, *LCD* 38 (2.3) y	6 months, FU 6 months post-intervention All arms used same delivery, i.e. group, but differed in intensity *CR* & *Control*: less than weekly *CR* + *EX* & *LCD*: staged approach (e.g., weekly > monthly)
Whitelock et al. 2019 [110]; United Kingdom; University	n = 107 *Intervention* 42.8 (10.5) y, 77.4% (F) *Control* 44.5 (10.7) y, 70.4% (F)	8 weeks, no FU Individual, weekly
Zwickert et al. 2016 [104]; Australia; University	n = 60 71.7% (F), 44.3 y (SD not reported)	Each arm had a different intervention duration and FU period *CBT* + *ITS*: 12 month, FU 3 months post-intervention *CBT* + *MTS*: 9 month, FU 6 months post-intervention Group, staged approach (e.g. weekly > monthly)

^*^Participant characteristics are provided for all participants in trial, or separately for each intervention arm, depending what data are reported in publications

^a^Four participants were randomized in error; *CBT-A* cognitive behavior therapy for weight loss with the addition of support people, *CBT + ITS* cognitive-behavior therapy plus intensive technological support, *CBT + MTS* cognitive-behavior therapy plus minimal technological support, *CBT-SP* cognitive behavior therapy for weight loss alone, *CER* continuous energy restriction, *CR* calorie restriction, *CR + EX* calorie restriction plus exercise, *FU* follow-up, *IER* intermittent energy restriction, *HC* higher carbohydrate diet, *HP* higher protein diet, *HPF* high-protein/lower fiber supplement shake, *LCD* low-calorie diet, *LPF* low-protein/fiber supplement shake, *MIC* motivational interviewing and internet condition, *NPC* nutrition psychoeducation and internet condition, *TRE* time-restricted eating, *UC* usual care, *USA* United States of America, *y* years, *8KKW* 8 kcal/kg of body weight/week, *20KKW* 20 kcal/kg of body weight/week

### Delivery features

The frequency of delivery features across interventions are reported in Table 2. Most interventions aimed for weight loss (k = 34 of 64, 53%) or weight loss and maintenance (k = 22 of 64, 34%). The most common psychological theory or frameworks underpinning interventions were, cognitive behavioral therapy (k = 24 of 64, 38%), and other general theory (k = 19 of 64, 30%). Almost all interventions targeted individuals (k = 63 of 64, 98%). Materials used to support behavior change included information sheets or booklets (k = 57 of 64, 89%) and diet monitoring materials (k = 46 of 64, 72%). Interventions were primarily delivered by a dietitian or nutritionist (k = 47 of 64, 73%) and/or psychologist or counsellor (k = 27 of 64, 42%). For outcomes, all 64 intervention arms measured weight (100%) and most included measurement of psychosocial or mental health outcomes (k = 52 of 64, 81%), eating behaviors (k = 52 of 64, 81%) or physical health outcomes (k = 44 of 64, 69%). Most interventions were delivered in-person (k = 58 of 64, 91%), directly to the individual (k = 45 of 64, 70%).

**Table 2:** Frequency of delivery features across intervention arms (k)

Cluster	Codes/components	Frequency in intervention arms (%) k = 64
Why—theory	Weight maintenance	7 (11)
	Weight loss	34 (53)
	Weight loss and maintenance	22 (34)
Psychological theory or framework	Cognitive behavior therapy (CBT)	24 (38)
	Enhanced Cognitive Behavior Therapy (CBT-E)	0 (0)
	Acceptance and Commitment therapy (ACT)	0 (0)
	Dialectical Behavior Therapy (DBT)	0 (0)
	Family-based therapy (FBT)	0 (0)
	Interpersonal Therapy (IPT) for binge eating	0 (0)
	Trauma-informed care	0 (0)
	Compassion focussed therapy	0 (0)
	Motivational interviewing	7 (11)
	Other general theory	19 (30)
Target population/recipient of the intervention—AGE GROUP	Adolescent	0 (0)
	Adult	64 (100)
Target population/recipient of the intervention—WEIGHT Category	Overweight (25–30 kg/m^2^)	42 (66)
	Obesity (30–40 kg/m^2^)	60 (94)
	Severe obesity (≥ 40 kg/m^2^)	51 (80)
Target population/recipient of the intervention—individual vs with support person/s	Individual	63 (98)
	Individual with support person	1 (2)
	Family or household-based treatment approach	0 (0)
What—materials	Information sheets/booklets	57 (89)
	Food	9 (14)
	Diet monitoring materials	46 (72)
	Supplements	4 (6)
	Meal replacement products	4 (6)
	Sports equipment	0 (0)
	Fitness/activity tracker	23 (36)
	Body scales	9 (14)
	Food scales	5 (8)
	Mobile app	6 (9)
	Website access (online resources)	11 (17)
	Social media	0 (0)
	Other materials	19 (30)
What—procedures	Nutrition education	56 (88)
	Energy prescription or target	31 (48)
	Physical activity education	46 (72)
	Exercise classes	9 (14)
	Psychological component	43 (67)
What—outcome measures	Weight/adiposity	64 (100)
	Physical health outcomes	44 (69)
	Psychosocial/mental health outcomes	52 (81)
	Eating behavior outcomes	52 (81)
	Individual weighing at visits	59 (92)
	Blind weighing at visits	5 (8)
	Group weighing	1 (2)
	Communication about the ability to decline weight or opt out of weighing during visits	9 (14)
Who provided—intervention delivered by (personnel, training, qualifications)	Dietitian/ nutritionist	47 (73)
	Nurse	5 (8)
	Exercise physiologist/Physiotherapist/Personal trainer/Other exercise professional	15 (23)
	Psychologist/counsellor	27 (42)
	Physician—pediatrician, GP, endocrinologist	10 (16)
	Pharmacist	0 (0)
	Researcher/non-health professional	16 (25)
	Self-delivered	6 (9)
	Other	15 (23)
Training for interventionist	Training for the interventionist	46 (72)
How—delivery mode	Face-to-face	58 (91)
	Computer/Web-based/Online	22 (34)
	Call (telephone, video)/SMS	30 (47)
	Printed material	42 (66)
	Individual	45 (70)
	Group	33 (52)
	Peer support	1 (2)
Where—intervention setting	Hospital outpatient	6 (9)
	Hospital inpatient	2 (3)
	University/Research centre	31 (48)
	Primary care	5 (8)
	Community	10 (16)
	School	0 (0)
	Household residence	0 (0)
	Virtual	16 (25)
	Commercial provider centres	0 (0)
	Workplace	0 (0)
Post-intervention support	Referral to other services	2 (3)
	Additional information provided	6 (9)
	Other	2 (3)
Tailoring	Was there an element of tailoring in the intervention?	37 (58)
Modifications	Was the intervention modified?	4 (6)
Fidelity	Were any intervention fidelity procedures (actual or planned) described?	54 (84)

Intervention duration and contact time were available for 46 of the 64 intervention arms (72%). Duration ranged from four to 104 weeks (median 27 weeks) and the number of contacts ranged from one to 365 (median 24.5). The total combined duration of contact with the personnel delivering the intervention ranged from 0.7 to 60 h (median 16.25 h). The frequency of contact varied, with most interventions using a staged approach (e.g. weekly to > monthly; k = 28 of 64, 44%), followed by once a week (k = 19 of 64, 30%).

### Intervention strategies

**Fig. 2:**
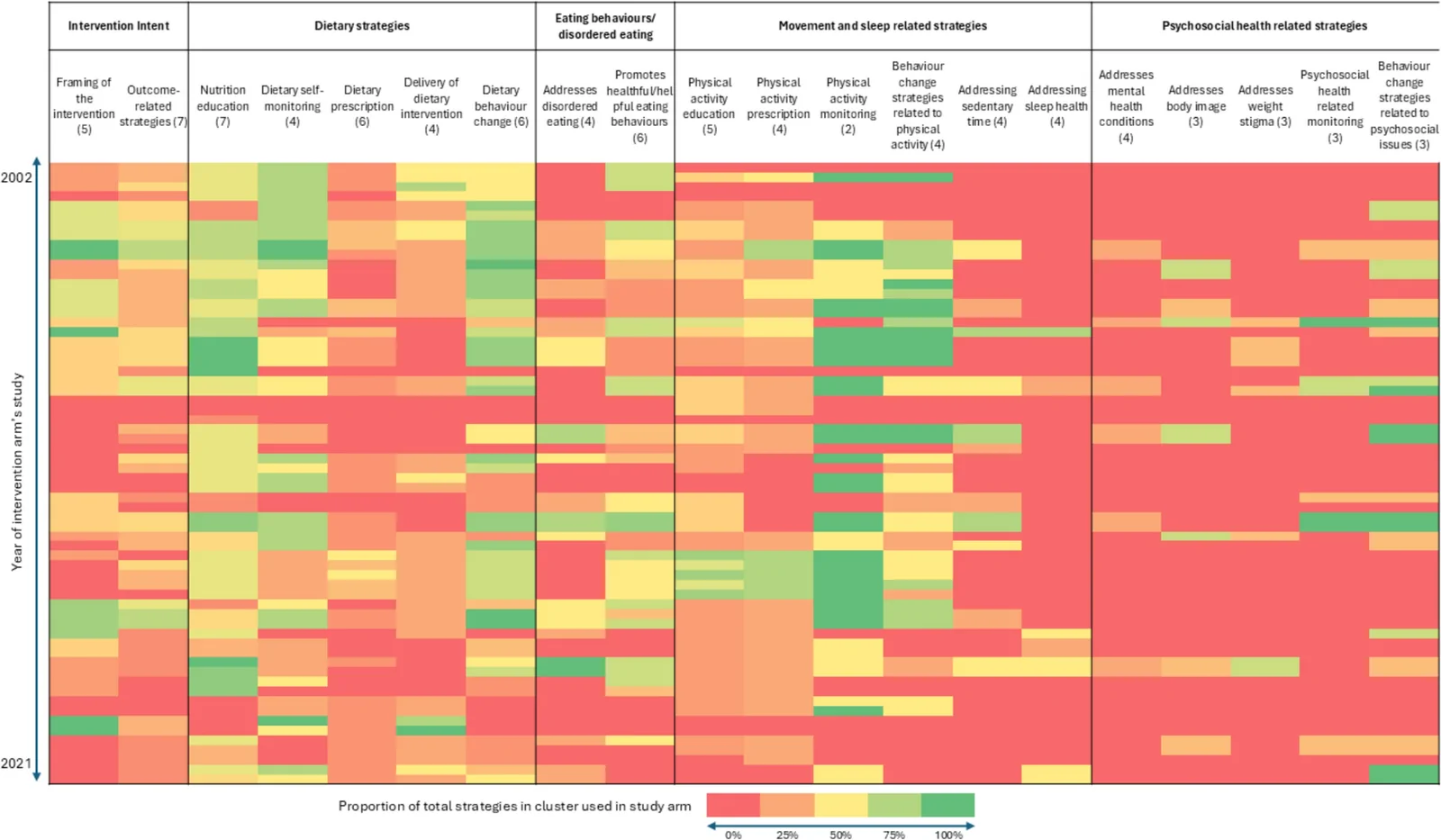
Frequency of intervention strategies across and within intervention arms. Each row represents an intervention arm arranged by year of the corresponding trial, starting from the oldest trial at the top down to the most recent trial (based on recruitment date, or publication date if unavailable). Each column represents clusters of intervention strategies within five broad categories; each number in brackets reports the number of strategies within that cluster. The colours of each square depict the proportion of total intervention strategies within that strategy cluster that are used in the intervention arm

Interventions used a mean (SD) of 24 (11.2) intervention strategies (Supplementary file 4). The heatmap in Fig. 2 shows the frequency of intervention strategies used within each intervention arm, grouped by strategy cluster and category. Within the five categories, most interventions included strategies within the *Dietary Strategies* (k = 62 of 64, 97%) category, the *Movement and Sleep Related Strategies* category (k = 57 of 64, 88%), and the *Eating Behavior and Disordered Eating* category (k = 47 of 64, 73%), with less than half of interventions including strategies from the *Psychosocial Health Related Strategies* category (k = 29 of 64, 45%). Across all clusters, most interventions included strategies from the *Nutrition Education* cluster (k = 58 of 64, 91%), the *Dietary Behavior Change* cluster (k = 54 of 64, 84%), *Physical Activity Education* cluster (k = 52 of 64, 81%) and the *Dietary self-monitoring* cluster (k = 51 of 64, 80%). The fewest number of strategies used in interventions were from the *Addresses weight stigma* (k = 8 of 64, 13%) and *Addressing sleep health* clusters (k = 10 of 64, 16%). The most common strategies within each category and cluster are described below.

#### Intervention intent category (two clusters)

The most common strategy within the *Framing of the Intervention (communications)* cluster was providing education that health behaviors are linked to health outcomes (k = 31 of 64, 48%) or providing education that weight loss is required to improve health outcomes (k = 29 of 64, 45%). Few interventions provided feedback on change in metabolic health outcomes (k = 8 of 64, 13%). Within the *Outcome Related Strategies* cluster, the most common strategies were weight focused goals (k = 39 of 64, 61%), provided feedback on weight change (k = 31 of 64, 48%) and encouraged self-monitoring of weight (k = 29 of 64, 45%). No interventions used weight loss rewards or incentives. Language used during interventions was mostly a combination of weight and health focused (k = 42 of 64, 66%), with fewer interventions using predominantly weight- (k = 14 of 64, 22%) or health- (k = 8 of 64, 13%) focused language alone.

#### Dietary strategies category (five clusters)

Within the *Nutrition Education* cluster, interventions mainly provided education on healthy eating (k = 51 of 64, 80%), portion sizes (k = 47 of 64, 73%) or macronutrient distribution (k = 44 of 64, 69%). Within the *Dietary Self-monitoring* cluster, the most common strategies were using food- (k = 35 of 64, 55%) or energy-based (k = 32 of 64, 50%) dietary self-monitoring and providing feedback (k = 34 of 64, 53%). Where a *Dietary Prescription* was used, this was often a hypocaloric diet (k = 34 of 64, 53%). Strategies to support *Delivery of the Dietary Intervention* included use of flexible (k = 36 of 64, 56%) or prescriptive meal plans (k = 13 of 64, 20%). Most frequently used *Dietary Behavior Change* strategies were problem solving (k = 45 of 64, 70%), goal setting (k = 42 of 64, 66%) and addressing the home food environment (k = 35 of 64, 55%). Few interventions used very low energy diets (k = 2 of 64, 3%), promoted the ad-libitum intake of ‘free foods’ (k = 3 of 64, 5%) or encouraged the use of meal replacement products (k = 4 of 64, 6%).

#### Eating behaviors/disordered eating category (two clusters)

Few interventions included strategies to address disordered eating behaviors (k = 21 of 64, 33%). A range of strategies were used to *Promote Healthful/Helpful Eating Behaviors* including promoting mealtime routines (k = 34 of 64, 53%) or encouraging mindful eating (k = 27 of 64, 42%). Few interventions provided education on the risk of eating disorders (k = 2 of 64, 3%) or encouraged intuitive eating practices (k = 8 of 64, 13%).

#### Movement and sleep strategies category (six clusters)

Most interventions (k = 52 of 64, 81%) included strategies within the *Physical Activity Education* cluster, with education to increase physical activity (k = 46 of 64, 72%) most common. *Physical Activity Prescription* included providing a flexible (k = 31 of 64, 48%) or prescriptive exercise plan (k = 20 of 64, 31%). Strategies used for *Physical Activity Monitoring* included self-monitoring (k = 42 of 64, 66%) with feedback provided (k = 27 of 64, 42%). Use of activity focused goals (k = 37 of 64, 58%) and problem-solving barriers to physical activity (k = 27 of 64, 42%) were the most frequently used within the cluster of *Behavior Change Strategies Related to Physical Activity. Addressed Sedentary Time* strategies included providing education to reduce/ limit sedentary time (k = 19 of 64, 30%) and including problem solving (k = 10 of 64, 16%). Few interventions included strategies for *Addressing Sleep Health* by providing education on sleep health (k = 10 of 64, 16%).

#### Psychosocial health strategies category (five clusters)

Overall, few strategies were used in this category. The most common strategy used for the *Addresses Mental Health Conditions* cluster was addressing self-esteem (k = 7 of 64, 11%). Four of the 64 interventions (6%) defined identifying mental health conditions as a specific intervention component. However, during verification meetings it was noted that many trials commonly identified mental health conditions and provided referral to appropriate support services, as part of routine clinical care. Strategies within the *Addresses Body Image* cluster mostly promoted body compassion, acceptance and/or positivity (k = 12 of 64, 19%). For the *Addresses Weight Stigma* cluster, strategies addressed weight-focused communication skills (k = 5 of 64, 8%) or provided education to increase resilience to weight stigma (k = 4 of 64, 6%). Self-monitoring of thoughts, feelings, and mood  (k = 7 of 64, 11%) was the most commonly used strategy within the *Psychosocial Health Related Monitoring* cluster. *Behavior Change Strategies Related to Psychosocial Health* frequently included skills to manage psychosocial health (k = 25 of 64, 39%) and goal setting (k = 12 of 64, 19%).

### Combinations of intervention strategy clusters

**Fig. 3:**
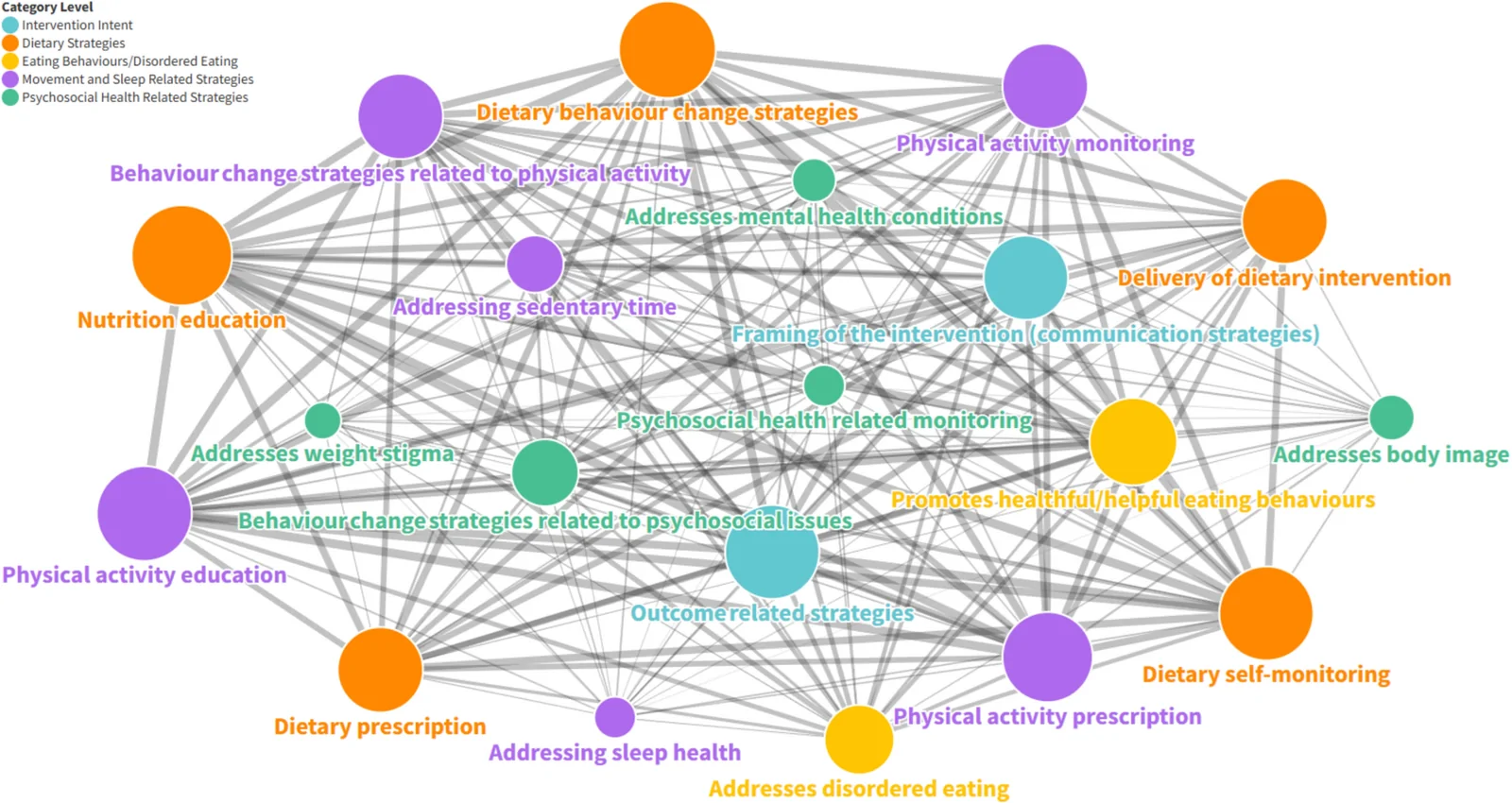
Frequency of individual and combinations of intervention strategy clusters across all interventions. Circles represent clusters of intervention strategy clusters with circle colours corresponding to category levels. Frequency of intervention strategy clusters are reflected by circle sizes with larger circles representing more common strategy clusters. Combinations and frequency strategy clusters are reflected by line width and connects, with thicker lines representing more common combinations

Figure 3 illustrates the frequency of individual and combinations of intervention strategy clusters across all interventions. Larger circles depict clusters with more commonly used strategies (e.g. see *dietary self-monitoring*), whereas smaller circles represent clusters with less frequently used strategies (e.g. see *addresses weight stigma*). The frequency of a combination of two clusters is depicted by the width of the line connecting them, with a thicker line representing increased occurrence. For example, interventions commonly had both nutrition education and dietary behavior change strategies, whereas nutrition education and psychosocial health-related monitoring did not frequently co-occur in interventions. The variations in combinations of clusters are portrayed through the number of connections between circles.

### Trialist verification of coding and sensitivity analyses

The percentage agreement in coding between the coding team and the codes verified by trial representatives was 85.3%. Changes in coding were often a result of intervention components being present in trials but not described in study publications, thus, these components were originally coded as not present. This commonly applied to intervention components that trialists considered not essential to publish as part of key distinguishable intervention features. For example, routine care procedures such as addressing mental and sleep health, or providing hardcopy leaflets were often not reported in publications but were revealed to be part of interventions through the verification process.

Sensitivity analyses of all eligible 139 intervention arms compared to verified intervention arms only (primary analysis) revealed lower frequency of most intervention components. (Supplementary files 4 and 5).

## Discussion

This is the first study to deconstruct and demonstrate the complexity of adult behavioral weight management interventions with eating disorder outcomes through detailed mapping of delivery features and intervention strategies. Overall, interventions were multi-faceted and used a range of strategies, most commonly from the clusters *nutrition education*, *dietary behavior change*, *dietary self-monitoring*, and *physical activity*. Assessing such detail is important as our findings highlight that although diet and physical activity strategies are central to weight management, the number and combinations of strategies vary greatly both across and within interventions. While our study demonstrates the complexity of weight management interventions through the variations and depth in the range of strategies used, it also identified less frequently used intervention components, particularly *psychological components*. Moreover, our coding verification procedures revealed differences in the detail of publicly *reported* trial procedures compared to those *implemented*. Thus, findings from this study have the potential to guide trialists in enhancing future intervention design and reporting, in addition to informing further research examining which intervention components may increase or decrease eating disorder risk.

Given the role of nutrition and physical activity in weight management, it is unsurprising that these were the most frequently used intervention components. *Nutrition education* strategies was the leading cluster, used by almost ninety percent of interventions, followed by the clusters *dietary behavior change*, *physical activity education*, and *dietary self-monitoring*. Apart from dietary self-monitoring, the most commonly used clusters of intervention strategies were consistent across both age groups, with an emphasis on improving diet and physical activity [18]. Research on behavior change techniques in adults has found that higher weight loss outcomes were linked to self-monitoring behaviors [122, 123], particularly calorie-counting [124], while feedback on outcomes and goal setting improved physical activity behaviors [125]. On average, adult interventions used fewer strategies per intervention arm compared to what we observed in our research on intervention components used in adolescent weight management interventions that measure eating disorder risk [18]. Adolescent interventions were more likely to focus on a healthy lifestyle, rather than weight loss alone. This may be due to developmental and psychological differences between adults and adolescents as well as interventions for adolescents having more of a family focus. Accordingly, strategies targeting sedentary behavior, weight loss incentives, cultural adaptations to diet, disordered eating, and meal time support were observed more frequently in adolescent interventions compared to adult interventions [18]. This suggests that adult interventions may offer less support compared to adolescent behavioral weight management.

The EDIT coding framework focused on intervention components that are important to consider when examining eating disorder risk [15, 16]. Those components that were infrequently used highlight potential gaps in intervention design for behavior weight management in the context of eating disorder risk. Clusters of strategies such as *addresses weight stigma*, *addresses sleep health*, *addresses mental health conditions*, *addresses body image* and *psychosocial health related monitoring* were observed in fewer than one-fifth of interventions. This aligns with qualitative evidence indicating that weight management interventions often provide insufficient support for mental health and emotional well-being [126]. In particular, addressing weight stigma, a commonly proposed eating disorder risk factor [127, 128], may help lower the likelihood of eating disorder development. Given the attention to these concepts has come to the forefront in recent years, we may have expected these strategies to be present in more recent studies. However, interventions including strategies for *addresses weight stigma*, *addresses mental health conditions*, *addresses body image* and *psychosocial health related monitoring* were more likely to be from studies published prior to 2016. The apparent reduced frequency of these intervention strategies over time may be due to resource constraints or incorporation in standard care and therefore are not protocolized procedures in publications.

Our coding verification procedures with trialists and sensitivity analyses also revealed discrepancies in the level of detail recorded in study protocols and publications, and what is provided during interventions and in clinical care. As such, the number of intervention components was lower in the sensitivity analyses when coding was based on published materials only and without trialist verification. Published manuscripts are often restricted in their descriptions of trial methods, in which case interpretations of intervention effects may be based on an incomplete understanding of implemented procedures. However, this information can be included in supplementary materials or trial registries. Recognizing gaps in intervention design as well as improving detailed reporting of intervention strategies has significant implications for the replication of trials, guideline development, clinical practice and understanding of risks and benefits. The EDIT framework [16] has the potential to be used as a comprehensive checklist of intervention components to support consistent reporting of trial procedures. Furthermore, it encourages the integration of eating disorder considerations in future interventions by increasing awareness of relevant components that may not be routinely described.

This study has several strengths. It is the first to map delivery features and intervention strategies used in adult behavioral weight management interventions measuring eating disorder outcomes, building on our earlier research in adolescents. A comprehensive search of clinical trial registries, including unpublished data, was conducted to identify eligible studies. The coding framework used to deconstruct intervention components was project specific [16]. It was informed by consultation with a range of stakeholders, including those with lived experience of eating disorders and higher weight [15]. Trialist verification of coding also revealed unpublished intervention components. The limitations of this study include the inclusion of behavioral weight management trials measuring at least one eating disorder outcome. This may represent interventions cognizant of eating disorders and may not be generalizable to all weight management interventions. Interventions delivered in other settings e.g. post bariatric surgery or aiming to treat co-existing conditions alongside overweight and obesity may include differing intervention components. The systematic search was completed in March 2022; while eligible ongoing trials were identified through trial registries and subsequently coded when protocols or manuscripts were published, all relevant trials to-date may not be included. The time since completing the search reflects the depth of work required to code and verify trials for this study. Most included trials were from English-speaking, high-income countries therefore may not reflect weight management trials in other global contexts. Many of the strategies assessed in this study overlap with a prior taxonomy of BCTs aimed at improving healthy eating and physical activity for weight loss [129]. While our coding framework considered and included common strategies to increase weight loss and improve metabolic health, those that may impact eating disorder risk were prioritized.

## Conclusion

Adult weight management interventions are commonly multifactorial and vary in nature, with nutrition and physical activity strategies most common. Despite the breadth and complexity of intervention strategies used, the prevalence of psychological and sleep related strategies was low, highlighting a potential gap in intervention design or reporting of intervention features. Our framework shows promise in guiding future intervention design by encouraging consideration of eating disorder risk and enhancing reporting of components of behavior weight management interventions. Moreover, our deconstruction of intervention components can be used in future studies to determine which components of weight management interventions may increase or decrease the development of eating disorder symptoms in high-risk individuals.

## Supplementary Information

Supplementary material 1.

## Data Availability

The dataset may be shared on request.
