# Supplementary material for: Unpacking weight management interventions measuring eating disorder risk in adults: coding of components of interventions in a systematic review
Source: J Eat Disord. 2026 Jun 17;14:202. doi: 10.1186/s40337-026-01667-x (PMC13520360; doi:10.1186/s40337-026-01667-x)
Supplement: Supplementary file 1 [file 40337_2026_1667_MOESM1_ESM.docx]

# SUPPLEMENTARY FILES

**Supplementary file 1:** Changes to the Protocol from Original Published Protocol

**Supplementary file 2:** Search String for each database

**Supplementary file 3:** EDIT Intervention Coding Guide

**Supplementary file 4:** Frequency of Intervention Strategies across intervention arms of verified trials (primary analyses) versus all eligible trials (sensitivity analyses)

**Supplementary file 5:** Frequency of delivery features of all eligible trials (sensitivity analysis)

## **Supplementary file 1: Changes to the Protocol from Original Published Protocol**

| **Number** | **Description** |
| --- | --- |
| **1** | Description of training for the interventionist added (10.2 Describe) |
| **2** | Extra option of ‘weekly’ added to intervention dose: intensity (13.4) |
| **3** | Post intervention support ‘Other’ added (14.3 Other) |
| **4** | Merged fidelity codes ‘planned fidelity measures’ and ‘actual fidelity measures’ together in one code (17.1 ‘Were any intervention fidelity procedures (actual or planned) described?’) |
| **5** | Addition of one intervention strategy, 18.6 ‘Language’- weight focused language or health focused language or a combination of weight and health focused language |

## **Supplementary file 2: Search String for each database**

**Database: Ovid MEDLINE(R) ALL**

Search Strategy:

--------------------------------------------------------------------------------

1 exp Obesity/

2 exp Overweight/

3 obes*.tw.

4 overweight.tw.

5 1 or 2 or 3 or 4

6 weight loss/

7 exp diet therapy/

8 exp bariatrics/

9 exp exercise/

10 anti-obesity agents/ or appetite depressants/

11 (diet* adj2 therap*).tw.

12 bariatric*.tw.

13 (low adj3 (energy or calor*) adj4 diet).tw.

14 ((pharma* or diet* or obes* or lifestyle or behavio*) adj3 (interven* or treat* or therap*)).tw.

15 ((calori* or diet*) adj3 (reduc* or restrict*)).tw.

16 (weight adj3 (manag* or los*)).tw.

17 (exercis* or physical activit*).tw.

18 HAES.mp.

19 health at every size.mp.

20 (weight adj2 neutral).mp.

21 nondiet.mp.

22 (non adj2 diet).mp.

23 (intuitive adj2 eat*).mp.

24 mindful*.tw. (9839)

25 6 or 7 or 8 or 9 or 10 or 11 or 12 or 13 or 14 or 15 or 16 or 17 or 18 or 19 or 20 or 21 or 22 or 23 or 24

26 Body Image/

27 (body adj3 (accept* or dissatisf* or image or satisf* or appreciat* or esteem)).tw.

28 "feeding and eating disorders"/ or anorexia nervosa/ or binge-eating disorder/ or bulimia nervosa/ or "feeding and eating disorders of childhood"/

29 (bulimi* adj3 symptom*).tw.

30 (disorder* adj3 eat*).tw.

31 (emotion* adj3 eat*).tw.

32 (diet* adj3 restr*).tw.

33 (binge adj3 eat*).tw.

34 extreme weight loss.tw.

35 loss of control.tw.

36 drive for thinness.tw.

37 ((weight or shape or eat*) adj3 concern).tw.

38 (eat* adj2 behavi*).tw.

39 26 or 27 or 28 or 29 or 30 or 31 or 32 or 33 or 34 or 35 or 36 or 37 or 38

40 randomized controlled trial/

41 (randomi?ed controlled trial* or RCT* or (controlled adj3 trial)).mp.

42 randomi?ed.ti.

43 clinical trials as topic.sh.

44 randomly.ab.

45 trial.mp.

46 clinical trial.mp.

47 40 or 41 or 42 or 43 or 44 or 45 or 46

48 5 and 25 and 39 and 47

***************************

RCT related key words

"clinical trials" OR "clinical trials as a topic" OR "randomized controlled trial" OR "Randomized Controlled Trials as Topic" OR "controlled clinical trial" OR "Controlled Clinical Trials as Topic" OR "random allocation" OR "randomly allocated" OR "allocated randomly" OR "Double-Blind Method" OR "Single-Blind Method" OR "Cross-Over Studies" OR "Placebos" OR "cross-over trial" OR "single blind" OR "double blind" OR "factorial design" OR "factorial trial" OR clinical trial* OR trial* OR rct* OR random* OR blind*

**Database: SCOPUS**

Obes* OR Overweight*

AND

“Weight loss” OR “Diet* OR bariatric* OR Exercis* OR “anti-obesity agent*” OR HAES OR “health at every size” OR “Weight neutral” OR “Intuitive eat*” OR Mindful*

((pharma* or diet* or obes* or lifestyle or behavio*) W/4 (interven* or treat* or therap*))

AND

“Body image*” OR “Eating disorder*” OR anorexia OR “binge-eating disorder*” OR bulimi* OR

“Emotion* eat*” OR Diet* restr*” OR “Binge eat*” OR "extreme weight loss*" OR “loss of Control” OR “Drive for thinness”

((weight or shape or eat*) W/3 concern*)

Scopus complete query

( TITLE-ABS-KEY ( "clinical trials" OR "clinical trials as a topic" OR "randomized controlled trial" OR "Randomized Controlled Trials as Topic" OR "controlled clinical trial" OR "Controlled Clinical Trials as Topic" OR "Clinical trial*" OR trial* OR rct OR random* ) ) AND ( ( TITLE-ABS-KEY ( obes* OR overweight* ) ) AND ( ( TITLE-ABS-KEY ( "Weight loss" OR diet* OR bariatric* OR exercis* OR "anti-obesity agent*" OR haes OR "health at every size" OR "Weight neutral" OR "Intuitive eat*" OR mindful* ) ) OR ( TITLE-ABS-KEY ( ( ( pharma* OR diet* OR obes* OR lifestyle OR behavio* ) W/4 ( interven* OR treat* OR therap* ) ) ) ) ) AND ( ( TITLE-ABS-KEY ( ( ( weight OR shape OR eat* ) W/3 concern* ) ) ) OR

( ( TITLE-ABS-KEY ( "Body image*" OR "Eating disorder*" OR anorexia OR "binge eating disorder*" OR bulimi* OR "Emotion* eat*" OR "Diet* restr*" OR "Binge eat*" OR "extreme weight loss*" OR "loss of Control" OR "Drive for thinness" ) ) OR ( TITLE-ABS-KEY ( ( ( weight OR shape OR eat* ) W/3 concern* ) ) ) ) ) )

**Database: Embase**

Search Strategy:

--------------------------------------------------------------------------------

1 obesity/

2 obes*.tw.

3 overweight.tw.

4 1 or 2 or 3

5 weight reduction/

6 diet therapy/ or diet restriction/ or low calory diet/ or low fat diet/

7 bariatric surgery/ or gastric banding/ or sleeve gastrectomy/

8 exercise/

9 antiobesity agent/

10 (diet* adj2 therap*).tw.

11 bariatric*.tw.

12 (low adj4 (energy or calor*) adj4 diet).tw.

13 ((pharma* or diet* or obes* or lifestyle or behavio*) adj3 (interven* or treat* or therap*)).tw.

14 ((calori* or diet*) adj3 (reduc* or restrict*)).tw.

15 (weight adj3 (manag* or los*)).tw.

16 (exercis* or physical activit*).tw.

17 HAES.mp.

18 health at every size.mp.

19 (weight adj2 neutral).mp.

20 nondiet.mp.

21 (non adj2 diet).mp.

22 (intuitive adj2 eat*).mp.

23 mindful*.tw.

24 5 or 6 or 7 or 8 or 9 or 10 or 11 or 12 or 13 or 14 or 15 or 16 or 17 or 18 or 19 or 20 or 21 or 22 or 23

25 body image/

26 (body adj3 (accept* or dissatisf* or image or satisf* or appreciat* or esteem)).tw.

27 eating disorder/ or anorexia nervosa/ or binge eating disorder/ or bulimia/

28 feeding behavior/

29 (bulimi* adj3 symptom*).tw.

30 (disorder* adj3 eat*).tw.

31 (emotion* adj3 eat*).tw.

32 (diet* adj4 restrain*).tw.

33 (binge adj3 eat*).tw.

34 extreme weight loss.tw.

35 loss of control.tw.

36 drive for thinness.tw.

37 ((weight or shape or eat*) adj3 concern).tw.

38 25 or 26 or 27 or 28 or 29 or 30 or 31 or 32 or 33 or 34 or 35 or 36 or 37

39 randomized controlled trial/ or controlled clinical trial/

40 (randomi?ed controlled trial* or RCT* or (controlled adj3 trial)).mp.

41 randomi?ed.ti.

42 randomly.ab.

43 trial.mp.

44 clinical trial.mp.

45 39 or 40 or 41 or 42 or 43 or 44

46 4 and 24 and 38 and 45

**Database: APA PsycInfo**

Search Strategy:

--------------------------------------------------------------------------------

1 Obesity/

2 Overweight/

3 obes*.tw.

4 overweight.tw.

5 1 or 2 or 3 or 4

6 weight loss/ or weight control/

7 diets/

8 exp bariatric surgery/

9 exp exercise/

10 (diet* adj2 therap*).tw.

11 bariatric*.tw.

12 (low adj3 (energy or calor*) adj4 diet).tw.

13 ((pharma* or diet* or obes* or lifestyle or behavio*) adj3 (interven* or treat* or therap*)).tw.

14 ((calori* or diet*) adj3 (reduc* or restrict*)).tw.

15 (weight adj3 (manag* or los*)).tw.

16 exercis*.mp. or physical activit*.tw.

17 HAES.mp.

18 health at every size.mp.

19 (weight adj2 neutral).mp.

20 nondiet.mp.

21 (non adj2 diet).mp.

22 (intuitive adj2 eat*).mp.

23 mindful*.tw.

24 6 or 7 or 8 or 9 or 10 or 11 or 12 or 13 or 14 or 15 or 16 or 17 or 18 or 19 or 20 or 21 or 22 or 23

25 Body Image/

26 (body adj3 (accept* or dissatisf* or image or satisf* or appreciat* or esteem)).tw.7088)

27 eating disorders/ or anorexia nervosa/ or binge eating disorder/ or bulimia/ or hyperphagia/ or "purging (eating disorders)"/

28 eating behavior/ or binge eating/ or dietary restraint/

29 (bulimi* adj3 symptom*).tw.

30 (disorder* adj3 eat*).tw.

31 (emotion* adj3 eat*).tw.

32 (diet* adj3 restr*).tw.

33 (binge adj3 eat*).tw.

34 extreme weight loss.tw.

35 loss of control.tw.

36 drive for thinness.tw.

37 ((weight or shape or eat*) adj3 concern).tw.

38 25 or 26 or 27 or 28 or 29 or 30 or 31 or 32 or 33 or 34 or 35 or 36 or 37

39 randomized controlled trials/ or clinical trials/ or randomized clinical trials/

40 (randomi?ed controlled trial* or RCT* or (controlled adj3 trial)).mp.

41 randomi?ed.ti.

42 randomly.ab.

43 trial.mp.

44 clinical trial.mp.

45 39 or 40 or 41 or 42 or 43 or 44

46 5 and 24 and 38 and 45

**************************

## **Supplementary file 3: EDIT Intervention Coding Framework**

Note: this coding framework (table 1 and figures 1-5) is adapted from previous versions published elsewhere (1, 2). It can be used for both adolescents and adults, therefore definitions provided in the framework are not limited to adults.

**Table 1. Delivery features**

| **Why – Theory** | | | | | | |
| --- | --- | --- | --- | --- | --- | --- |
| 1.1 | Weight maintenance | | | Intervention focuses on/aims for weight maintenance but not weight loss. For example, the intervention is designed to prevent further weight gain or keep participants at designated weight or BMI z-score. Weight loss or decrease in body mass is not the target. | | |
| 1.2 | Weight loss | | | Intervention focuses on decreases in body mass. For example, implementing specific diets / intervention strategies to induce weight loss or decreases in body mass or decrease BMI z-score. Although weight maintenance may be a focus following the initial period of weight loss the target of the intervention is first to decrease body mass. | | |
| 1.3 | Weight loss and maintenance | | | Intervention has two parts, each with a focus on decreases in body mass AND weight maintenance. For example, implementing specific diets / intervention strategies to induce weight loss or decreases in body mass or decrease BMI z-score. Weight maintenance must be a focus at some stage of the intervention following the initial period of weight loss or decrease in body mass as the target of the intervention. | | |
| **Psychological theory or framework underpinning intervention** | | | | | | |
| 2.1 | | | Cognitive behaviour therapy (CBT) | | |  |
| 2.2 | | | Enhanced Cognitive Behaviour Therapy (CBT-E) | | |  |
| 2.3 | | | Acceptance and Commitment therapy (ACT) | | |  |
| 2.4 | | | Dialectical Behaviour Therapy (DBT) | | |  |
| 2.5 | | | Family-based therapy (FBT) | | | Do not code family-based treatment of childhood obesity. |
| 2.6 | | | Interpersonal Psychotherapy (IPT) for binge eating | | |  |
| 2.7 | | | Trauma-informed care | | |  |
| 2.8 | | | Compassion focussed therapy | | |  |
| 2.9 | | | Motivational interviewing | | |  |
| 2.10 | | | Other General theory | | | Extract theory name reported in intervention. For example, general behaviour change theories such as Social Cognitive Theory, Theory of Planned Behaviour, Health Belief Model, or intervention design frameworks such as intervention mapping, Behaviour Change Wheel. |
| **Target population/ recipient of the intervention – AGE GROUP** | | | | | | |
| 3.1 | | Adolescent | | | As reported by the trial, or when participants are aged 10-18 years. | |
| 3.2 | | Adult | | | As reported by the trial, or when participants are aged ≥19 years. | |
|  | | *Extracted except* | | | *Extract trial reported age range.* | |
| **Target population/ recipient of the intervention – WEIGHT Category** | | | | | | |
| 4.1 | | Overweight | | | As reported by the trial, or when participants have a BMI ≥25 kg/m^2^ – 30 kg/m^2^ or BMI z-score of 1-2 or between the 85th-95th BMI percentile. | |
| 4.2 | | Obesity | | | As reported by the trial, or when participants have a BMI ≥30kg/m^2^ – 40 kg/m^2^ or BMI z-score ≥2 or ≥95th BMI percentile. | |
| 4.3 | | Severe Obesity | | | As reported by the trial, or when participants have a BMI ≥40kg/m^2^ or BMI z-score of 3 or ≥99th BMI percentile. Note: in older trials this may be reported as morbid obesity.  Note: if states obesity and doesn’t provide an upper cut off, assume this also includes severe obesity. | |
|  | | Extract | | | Code weight target category as per trial reported category and extract trials definition for each category as relevant. | |

| **Target population/ recipient of the intervention – Individual vs with support person/s *(code only 1 recipient)*** | | |
| --- | --- | --- |
| 5.1 | Individual | A person with overweight or obesity participates in the intervention by themselves. |
| 5.2 | Individual with support person | For example, parent(s) complete intervention with participant (family-based treatment approach), or partners or friend attends the intervention with the participant. Do not code if adolescents require parental attendance to attend meetings due to age, without a family-based approach to the intervention. |
| 5.3 | Family or household-based treatment approach | Family/household approach is taken to treatment, e.g. whole of family-oriented changes to diet/activity. |

| **What – materials** | | | | | | | | |
| --- | --- | --- | --- | --- | --- | --- | --- | --- |
| 6.1 | | | Information sheets/ booklets | | | | This includes any type of written or visual printed materials (includes images). For example, fact sheets, newsletters, recipes, meal or exercise plans etc. This can include hardcopy (e.g. physical pamphlet) or softcopy (e.g. email) resources. Assumption that information sheets/ booklets are included if personalised diet plan or similar is provided. | |
| 6.2 | | | Food | | | | This includes fresh foods, portioned meals and meal-ordering services (e.g. Light and Easy, Jenny Craig), provided as part of the intervention. Do not code the recommendation to eat certain foods if participants are required to purchase food items themselves, or if catering is provided for a session. Explicit statement that food is provided is required to code. | |
| 6.3 | | | Diet monitoring materials | | | | This includes any type of diet monitoring materials that are provided (e.g. checklists, food diary, calorie counting). | |
| 6.4 | | | Supplements | | | | This includes vitamins, protein powders, and other supplements to add to meals.  Do not code the recommendation to purchase supplements, if participants are required to purchase items themselves. | |
| 6.5 | | | Meal replacement products | | | | This includes any products used to substitute a standard meal, such as shakes, bars, meal replacement powders, provided as part of the intervention.  Do not code the recommendation to purchase and use meal replacement products, if participants are required to purchase items themselves. | |
| 6.6 | | | Sports equipment | | | | This includes exercise bands, weights, treadmill etc, provided to participants as part of the intervention. Do not code the recommendation to purchase sports equipment, if participants are required to purchase items themselves. | |
| 6.7 | | | Fitness/ activity tracker | | | | This includes physical pedometers or accelerometers provided to participants as part of the intervention to track activity. Includes paper-based tracking/diaries. Do not code the recommendation to purchase and use activity trackers, if participants are required to purchase items themselves, or if solely used as a data collection method. | |
| 6.8 | | | Body scales | | | | This includes ‘bathroom’ scales to self-monitor weight at home. Do not code the recommendation to purchase and use scales, if participants are required to purchase items themselves, or if solely used as a data collection method. | |
| 6.9 | | | Food scales | | | | This includes ‘food’ scales to measure food portions at home.    Do not code the recommendation to purchase and use scales, if participants are required to purchase items themselves. | |
| 6.10 | | | Mobile app | | | | This includes mobile apps for the intervention and used with participants as part of the intervention. Where possible note if the app was interactive/2-way communication, versus 1-way information delivery. This can include the use of activity tracker mobile applications. | |
| 6.11 | | | Website access (online resources) | | | | This includes websites specific for the trial and used with participants as part of the intervention. Where possible note if the website was interactive/2-way communication, versus 1-way information delivery.   Do not code if a website is only used for participant recruitment or to share trial materials after the study. | |
| 6.12 | | | Social media | | | | This includes any social media (e.g. Facebook) pages, groups or channels specific for the trial and used with participants as part of the intervention. Do not code if social media is only used for participant recruitment or to share trial materials after the study. | |
| 6.13 | | | Other materials | | | | Please describe any other materials provided to participants as part of the intervention that are not captured in the above items. | |
| **What – procedures** | | | | | | | | |
| *Note this delivery feature category provides a high-level coding of the types of intervention content, specific details of the intervention content will be coded by the Intervention Strategies.* | | | | | | | | |
| 7.1 | Nutrition education | | | | This includes information on food groups, daily food intakes, portion sizes, eating patterns, improving diet quality and nutrition knowledge, etc. For example, written resource provided listing servings in approximately 100 kcal portions or when dietitian reviews dietary intake, eating behaviours, and encourages adherence to the intervention. | | | |
| 7.2 | Energy prescription or target | | | | This includes utilising energy-based diets (e.g. Intermittent Energy Restriction) to energy intake targets to promote decrease in body mass or assist in weight maintenance. | | | |
| 7.3 | Physical activity education | | | | This includes information on physical activity benefits, how to schedule and perform activity. | | | |
| 7.4 | Exercise classes | | | | This includes practical gym classes, group activity sessions, individual personal training/ exercise physiology sessions etc, delivered as part of the intervention. | | | |
| 7.5 | Psychological component | | | | This includes information or education about psychological health, counselling and/or use of psychological techniques (Cognitive Behaviour Therapy, anticipatory guidance etc). | | | |
| **What – outcome measures** | | | | | | | | |
| 8.1 | Weight/ adiposity | | | | This includes measurement of weight, waist circumference, body composition etc. | | | |
| 8.2 | Physical health outcomes | | | | This includes measurement of a range of physical health outcomes, e.g. blood pressure, blood test, fitness. | | | |
| 8.3 | Psychosocial /Mental health outcomes | | | | This includes measurement of any psychosocial or mental health outcomes, e.g. depression, quality of life, self-esteem, anxiety, body image, weight-bias internalisation. Do not code eating disorder outcome measures as trials were required to include an eating disorder outcome measure to be eligible to join the EDIT Collaboration. | | | |
| 8.4 | Eating behaviour outcomes | | | | This refers to eating behaviours or appetite-related outcomes (e.g. DEBQ, TFEQ). Do not code eating disorders or disordered eating behaviours (e.g. binge eating, EDE-Q, CHEAT, EAT, EDI). | | | |
| *If measuring weight:* | | | | | | | | |
| 8.5 | Individual weighing at visits | | | | This includes any weighing that is performed in private in an intervention, e.g. participant measured at the beginning of each one-on-one dietary counselling session. Assumption that individual weighing is conducted unless it is stated clearly otherwise. | | | |
| 8.6 | Blind weighing at visits | | | | This includes any time a participant is weighed with the measure recorded without sharing the weight measure with the participant.  This does not refer to trials having a blinded outcome assessor. | | | |
| 8.7 | Group weighing | | | | This includes weights measured in a group context of more than one participant. | | | |
| 8.8 | Communication about the ability to decline weight or opt out of weighing during visits | | | | This includes any written or verbal communication with participants that weighing was voluntary, and they could choose to opt-out at any time. | | | |
| **Who provided – intervention delivered by (personnel, training, qualifications)** | | | | | | | | |
| 9.1 | Dietitian/ nutritionist | | | | This includes research or student dietitians. | | | |
| 9.2 | Nurse | | | | This includes research or student nurses. | | | |
| 9.3 | Exercise Physiologist/ Physiotherapist/ Personal trainer/ Other exercise professional | | | | This includes research or student exercise professionals. | | | |
| 9.4 | Psychologist/ counsellor | | | | This includes research or student psychologists. | | | |
| 9.5 | Physician – pediatrician, GP, endocrinologist | | | | This includes any medical specialists and research or student physicians. | | | |
| 9.6 | Pharmacist | | | | This includes research pharmacists. | | | |
| 9.7 | Researcher/ non-health professional | | | | This includes researchers or individuals who do not have a health professional background. | | | |
| 9.8 | Self-delivered | | | | No researcher/health professional contact during the intervention (e.g. self-help program, application, website-based intervention).  Excluding a once-off session/contact to explain the self-delivered content. | | | |
| 9.9 | Other, free text | | | | Please describe other professionals who are involved in the intervention delivery not listed above (e.g. school-teacher). | | | |
| **Training for interventionist** | | | | | | | | |
| 10.1 | | | Training for the interventionist | | | | Was specific training provided to intervention facilitators? | |
| 10.2 | | | Describe | | | | If yes, briefly describe the type and duration of the training. | |
| **How – delivery mode** | | | | | | | | |
| 11.1 | | Face-to-face | | | | In person delivery. | | |
| 11.2 | | Computer/ Web-based / Online | | | | Website, mobile application, emails etc. | | |
| 11.3 | | Call (telephone, video)/ SMS | | | | Any form of phone or video calls including appointments, follow up phone calls/texts, text-based intervention delivery. | | |
| 11.4 | | Printed material | | | | Handouts, workbooks, meal plans, recipes etc. | | |
| *In additional to the above coding, provide the summary level coding of the intervention delivery.* ***Code all that apply****, for example there may be initial individual session, followed by a series of group sessions.* | | | | | | | | |
| 11.5 | | Individual | | | | Delivery on individual / one-on-one basis, e.g. individual person, family (parent/child). Assumption that the delivery mode is individual if not stated otherwise. | | |
| 11.6 | | Group | | | | Delivery is to a group (any sessions with more than one participant attending).  Note: this can include any aspect that is delivered to a group of participants including online forums. | | |
| 11.7 | | Peer support | | | | Intervention delivery to the participant who has a peer support worker as part of the intervention (i.e. intervention links the participant with a peer support worker).  Do not code if participant is encouraged to bring a support person to the intervention (i.e. code 5.2) | | |
| **Where – intervention setting** | | | | | | | | |
| *Refers to where the clinician and participant meet or interact. Do not code intervention setting if only used for recruitment, e.g. participants recruited through hospital inpatient setting but then all intervention sessions were delivered through outpatient setting.  Author location or address cannot be used to determine intervention setting.* ***Code all setting relevant for the participant intervention setting.*** | | | | | | | | |
| 12.1 | | | | Hospital outpatient | | | | Attending a hospital facility as an outpatient for the intervention (clinic, university hospital etc). |
| 12.2 | | | | Hospital inpatient | | | | Participants receiving intervention components while an inpatient in a hospital setting. |
| 12.3 | | | | University / research centre | | | | Any university or research institute setting. |
| 12.4 | | | | Primary care | | | | Any primary health care setting such as GP clinic, medical centre etc. |
| 12.5 | | | | Community | | | | Any community-based settings, such as community centres, support groups, local government etc. |
| 12.6 | | | | School | | | | Any formal education setting such as primary school or high school, including after hours care settings. |
| 12.7 | | | | Household residence | | | | Any component of the intervention that is delivered in a participant’s home, such as home visits. |
| 12.8 | | | | Virtual | | | | Receiving intervention through computer/mobile app/online or call/SMS components. |
| 12.9 | | | | Commercial provider centres | | | | Any commercial provider offices such as Weight Watches, Jenny Craig, Slimming World etc where participants attend the setting. |
| 12.10 | | | | Workplace | | | | Any workplace that the intervention is delivered via, e.g. corporate programs, military. |
| **When and how much (intervention dose)** | | | | | | | | |
| 13.1 | | | Total number of contacts | | | | Record total number of intervention sessions during intervention (not follow-up period). Record number and duration of contacts in extracted excerpts. | |
| 13.2 | | | Overall intervention duration (weeks) | | | | Total length of the intervention period (in weeks). The intervention period is defined as the time when any contact/ advice is provided by study personnel and includes weight loss and weight maintenance periods but excludes follow-up period where there is no ongoing contact with the study team (excluding solely data collection). ‘Keeping in touch’ strategies such as newsletters to encourage retention at follow-up, but that do not provide advice, will not be considered part of the intervention. | |
| 13.3 | | | Total duration of contacts/sessions (minutes) | | | | Sum of duration of all intervention sessions. Select unclear if no information provided or if intervention does not have any verbal form of contact with participants (e.g. text-messaging, online video education only interventions). Document anything that is reported re: time.    *Note: when unclear is selected – check with Trialist to see if they can provide an estimate.* | |
| 13.4 | | | Intensity | | | | This relates to the frequency of the intervention sessions, code as less than weekly, weekly, every 2-3 weeks, monthly, >monthly, staged approach (weekly to >monthly).    *Note: select only one, i.e. if different frequencies select staged approach.* | |
| **Post-intervention support** | | | | | | | | |
| 14.1 | | | Referral to other services | | | | At the end of the end of the intervention (i.e., end of contact with study personnel for intervention, not data collection), participants are referred to other appropriate services (e.g. referred to local dietitians for ongoing support). | |
| 14.2 | | | Additional information provided | | | | At the end of the end of the intervention (i.e. end of contact with study personnel for intervention, not data collection period), participants are provided with addition information (verbal or written) (e.g. advice on healthy eating). | |
| 14.3 | | | Other | | | | At the end of the intervention (i.e. end of data collection period), is there any other support providing (other than referrals to other services or additional information). | |
| **Tailoring** | | | | | | | | |
| 15.1 | | | Was there an element of tailoring in the intervention? | | | | Tailoring refers to if the intervention was planned to be personalised, titrated or adapted at the participant level. For example, a personalised meal plan to suit the individuals lifestyle and dietary preferences. If tailoring at the participant level was planned code as yes and briefly describe / extract text explaining how the intervention will be/was tailored. Do not code if energy levels are based on age/sex, or if the intervention only includes individual delivery or goal setting.    *Note: only code if the trial explicitly states personalisation or tailoring.* | |
| 15.2 | | | Who or which subgroup was the intervention tailored to? | | | | Record if the tailoring was for ‘all’ participants, or to certain subgroup (e.g. only those rated as having low for self-esteem received a personalised self-efficacy package). | |
| **Modifications** | | | | | | | | |
| 16.1 | | | Was the intervention modified? | | | | Modifications refers to if the intervention was modified during the study at the intervention level, i.e. changed from what was planned in the intervention protocol. This may have been due to changes required with COVID-19 restrictions or a range of other reasons. If the intervention was modified at the intervention level code as yes and briefly describe / extract text explaining how the intervention was modified. Modifications will not be reported in published trial protocols.    The ‘Intervention Coding Request Form’ will ask Trial Representative to reported modifications to the intervention; this will be used to cross-check modifications coded from the materials received. | |
| **Fidelity** | | | | | | | | |
| 17.1 | | | Were any intervention fidelity procedures (actual or planned) described? | | | | Planned fidelity relates to proactive steps to ensure and/or monitor fidelity of the intervention; this may be described in the trial protocol as it relates to the planned processes/methods that will be used. For example, standardised training and facilitator manuals, weekly facilitator meetings, independent observations of intervention delivery, checklist of key messages delivered. The key to planned fidelity is the intentional purpose to ensure the intervention is delivered consistently as it was developed to be delivered.    Actual fidelity relates to measures / outcomes of fidelity reported based on the actual delivery of the intervention, for example the number of sessions attended by all participants. Actual fidelity will not be reported in published trial protocols. For example, rating by an independent experienced observer using a standardised process found that all intervention messages were consistently delivered to participants. This may not be reported in detail, a results paper may report that the planned activities to ensure fidelity were completed but not report the findings or outcomes of that. | |

 **Figure 1. Intervention intent category, clusters, and strategies** **Figure 2. Dietary strategies category, clusters, and strategies**

 **Figure 3. Eating behaviors/disordered eating category, clusters, and strategies** **Figure 4. Movement and sleep related strategies category, clusters, and strategies** **Figure 5. Psychosocial health related strategies category, clusters, and strategies**

## **Supplementary file 4: Frequency of Intervention Strategy Components across intervention arms (k) of verified trials (primary analyses) and all eligible trials (sensitivity analyses)**

Primary analyses included trials with intervention arms that were verified by trialists. Of the 58 eligible trials, 26 trials with 64 intervention arms were verified and included in the primary analyses. Sensitivity analyses consisted of all 58 eligible studies with 139 intervention arms (k). This included original coding of all studies in primary analyses as well as 26 additional studies that could not be verified.

| **Cluster** | **Codes/components** | **Frequency in *verified* intervention arms (%)**  **(Primary analyses, k=64)** | **Frequency in *all eligible* intervention arms (%)**  **(Sensitivity analyses,  k =139)** |
| --- | --- | --- | --- |
| **Intervention Intent, Framing and Outcomes category** | | |  |
| Framing of the intervention (communication strategies) | Education provided on obesity as a disease | 17 (27) | 0 (0) |
|  | Education that weight loss is required to improve health outcomes | 29 (45) | 8 (6) |
|  | Education that health outcomes are not dependent on weight | 17 (27) | 5 (4) |
|  | Education that health behaviours are linked to health outcomes | 31 (48) | 5 (4) |
|  | Feedback on change in metabolic health outcomes (e.g. insulin sensitivity, cholesterol levels) | 8 (13) | 3 (2) |
|  | **Interventions with any strategy in this cluster** | **41 (64)** | **14 (10)** |
| Outcome related strategies | Encourages weight focused goals | 39 (61) | 55 (40) |
|  | Discourages weight-focused goals (instead focused on health-related goals) | 4 (6) | 1 (1) |
|  | Feedback on weight change during the intervention | 31 (48) | 22 (16) |
|  | Feedback on other measures of weight adiposity (e.g. body composition, waist circumference) | 9 (14) | 8 (6) |
|  | Promotes weight loss rewards or incentives | 0 (0) | 0 (0) |
|  | Encourages self-monitoring of weight (e.g. self-weighing at home) | 29 (45) | 32 (23) |
|  | Discourages home weighing or frequent weighing | 10 (16) | 4 (3) |
|  | **Interventions with any strategy in this cluster** | **51 (80)** | **69 (50)** |
| **Dietary strategies category** | | |  |
| Nutrition Education | Education on portion size (e.g. portion plate model, serve sizes, etc) | 47 (73) | 32 (23) |
|  | Education on label reading | 38 (59) | 19 (14) |
|  | Education on metabolism | 21 (33) | 6 (4) |
|  | Education on healthy eating guide (e.g. promotes balanced meals and food groups) | 51 (80) | 39 (28) |
|  | Education on energy/ macronutrient (e.g. fat, sugar) content of foods | 44 (69) | 33 (24) |
|  | Categorisation of foods   as good versus bad (e.g. traffic light system; defines foods as good vs bad (e.g treat/ sometimes foods) | 23 (36) | 12 (9) |
|  | Provides cultural adaptations relating to diet | 10 (16) | 0 (0) |
|  | **Interventions with any strategy in this cluster** | **58 (91)** | **56 (40)** |
| Dietary self-monitoring | Dietary self-monitoring – food based (e.g. food diary, points system) | 35 (55) | 27 (19) |
|  | Dietary self-monitoring – energy based (e.g. calorie counting) | 32 (50) | 48 (35) |
|  | Dietary self-monitoring – weighing food | 14 (22) | 9 (7) |
|  | Review/feedback on self-monitoring (e.g. feedback on food diary) | 34 (53) | 28 (20) |
|  | **Interventions with any strategy in this cluster** | **51 (80)** | **67 (48)** |
| Dietary prescription | Hypocaloric diet (reduced calorie diet) | 34 (53) | 52 (37) |
|  | Traffic light diet (categorising foods as red, yellow, green) | 7 (11) | 5 (4) |
|  | Intermittent energy restriction/ intermittent fasting (chrononutrition) | 7 (11) | 6 (4) |
|  | Macronutrient prescription (e.g. low-carbohydrate, high-protein) | 5 (8) | 7 (5) |
|  | Ketogenic diet | 0 (0) | 0 (0) |
|  | Very low energy diet (VLED/ VLCD - restrictive calorie restriction e.g. 800-1000 kcal/ day) | 2 (3) | 7 (5) |
|  | **Interventions with any strategy in this cluster** | **42 (66)** | **63 (45)** |
| Delivery of dietary intervention | Prescriptive/ specific meal plan (external control) | 13 (20) | 23 (17) |
|  | Flexible meal plan (provides choice, ownership over dietary intake) | 36 (56) | 33 (24) |
|  | Use of meal replacement products – partial or full | 4 (6) | 9 (7) |
|  | Promotes ‘free’ foods, ad-lib intake of certain foods | 3 (5) | 8 (6) |
|  | **Interventions with any strategy in this cluster** | **42 (66)** | **54 (39)** |
| Dietary behaviour change strategies | Problem solving barriers to dietary change | 45 (70) | 65 (47) |
|  | Feedback on dietary behaviours (e.g. diet history at visits) | 30 (47) | 27 (19) |
|  | Encourages dietary focused goals with/ without review | 42 (66) | 59 (42) |
|  | Shopping support (planning, product choice, family/ partner involvement in food purchases) | 15 (23) | 19 (14) |
|  | Addresses home/food environment (e.g. identifying triggers, permissive vs restrictive environment; stimulus control) | 35 (55) | 43 (31) |
|  | Addresses food/meal preparation skills (e.g. cooking demonstrations, recipes) | 23 (36) | 25 (18) |
|  | **Interventions with any strategy in this cluster** | **54 (84)** | **91 (66)** |
| **Eating behaviours/disordered eating category** | | |  |
| Addresses disordered eating | Identifies disordered eating behaviours (e.g. binge eating, emotional eating, secret eating, guilt related to eating, loss of control over eating) | 18 (28) | 6 (4) |
|  | Explores individual underlying causes/ drivers of disordered eating (e.g. teasing/ bullying, trauma, body image disturbance/pre-occupation with weight and shape, emotional regulation) | 9 (14) | 2 (1) |
|  | Addresses disordered eating behaviours and cognitions (e.g. identifying triggers, strategies to prevent emotional eating, over-focus on energy expenditure) | 21 (33) | 14 (10) |
|  | Education on risk of eating disorders | 2 (3) | 0 (0) |
|  | **Interventions with any strategy in this cluster** | **28 (44)** | **17 (12)** |
| Promotes healthful/helpful eating behaviours | Promotes mealtime routines (e.g. regular meals, avoid meal skipping) | 34 (53) | 16 (12) |
|  | Promotes meal time support (e.g. support while eating, family meals, social eating) | 17 (27) | 16 (12) |
|  | Addresses meal time environment | 13 (20) | 8 (6) |
|  | Increasing awareness of hunger/ fullness/ satiety | 23 (36) | 25 (18) |
|  | Encourages mindful eating principles or practice (e.g. avoiding distractions while eating) | 27 (42) | 29 (21) |
|  | Encourages intuitive eating principles or practice (e.g. Promotes anti-diet, hunger and fullness, food enjoyment, body respect) | 8 (13) | 7 (5) |
|  | **Interventions with any strategy in this cluster** | **44 (69)** | **45 (32)** |
| **Movement and sleep related strategies category** | | |  |
| Physical activity education | Education to increase physical activity (e.g. staged introduction of activity, suggested activities) | 46 (72) | 64 (46) |
|  | Promotes joyful movement and activity | 23 (36) | 16 (12) |
|  | Encourages strict/ formal activity plan (e.g. gym program) | 7 (11) | 25 (18) |
|  | Education on Non Exercise Activity Thermogenesis (NEAT) (energy expended during tasks of daily living) | 7 (11) | 5 (4) |
|  | Provides cultural adaptations relating to physical activity | 0 (0) | 0 (0) |
|  | **Interventions with any strategy in this cluster** | **52 (81)** | **79 (57)** |
| Physical activity prescription | Provides a prescriptive exercise plan | 20 (31) | 44 (32) |
|  | Provides flexible exercise plan (e.g. suggested activities, encouraging choice) | 31 (48) | 21 (15) |
|  | Provides supervised group exercise classes/ program | 9 (14) | 21 (15) |
|  | Provides individual personal training | 6 (9) | 6 (4) |
|  | **Interventions with any strategy in this cluster** | **47 (73)** | **67 (48)** |
| Physical activity monitoring | Self-monitoring of activity (e.g. diary, pedometer) | 42 (66) | 55 (40) |
|  | External feedback on self-monitoring (e.g. feedback on exercise diary, step count) | 27 (42) | 21 (15) |
|  | **Interventions with any strategy in this cluster** | **42 (66)** | **55 (40)** |
| Behaviour change strategies related to physical activity | Encourage activity focused goals (including time, duration, mode) with/ without review | 37 (58) | 49 (35) |
|  | Increasing skills to undertake physical activity (e.g. demonstration of activity such as pictures/ videos/ live demos, help with scheduling/ planning for activity) | 20 (31) | 18 (13) |
|  | Feedback on physical activity behaviours and/ or change in fitness | 19 (30) | 8 (6) |
|  | Problem solving barriers to physical activity | 27 (42) | 28 (20) |
|  | **Interventions with any strategy in this cluster** | **42 (66)** | **61 (44)** |
| Addressing sedentary time | Education to reduce/ limit sedentary time (e.g. screen time) | 19 (30) | 7 (5) |
|  | Sedentary time focused goals (time, duration, mode) with/ without review | 7 (11) | 2 (1) |
|  | Encourages self-monitoring of sedentary time (e.g. screen time monitoring, setting app limits, reminders to stand) | 0 (0) | 0 (0) |
|  | Problem solving barriers to reducing sedentary time | 10 (16) | 3 (2) |
|  | **Interventions with any strategy in this cluster** | **19 (30)** | **7 (5)** |
| Addressing sleep health | Education on sleep health (e.g. duration, quality, routine) | 10 (16) | 8 (6) |
|  | Sleep health focused goals with/without review | 1 (2) | 0 (0) |
|  | Encourages self-monitoring of sleep (e.g. sleep diary) | 3 (5) | 0 (0) |
|  | Problem solving barriers to improving sleep health | 3 (5) | 2 (1) |
|  | **Interventions with any strategy in this cluster** | **10 (16)** | **8 (6)** |
| **Psychosocial health related strategies category** | | |  |
| Addresses mental health conditions | Identifies mental health condition | 4 (6) | 0 (0) |
|  | Provides referral for psychological support | 0 (0) | 0 (0) |
|  | Addresses mental health condition within the intervention | 0 (0) | 0 (0) |
|  | Addresses self-esteem | 7 (11) | 2 (1) |
|  | **Interventions with any strategy in this cluster** | **11 (17)** | **2 (1)** |
| Addresses body image | Addresses body image concerns | 6 (9) | 12 (9) |
|  | Education on the role of social media (e.g. media literacy training) | 0 (0) | 1 (1) |
|  | Promotes body compassion/ acceptance/ positivity | 12 (19) | 9 (7) |
|  | **Interventions with any strategy in this cluster** | **12 (19)** | **15 (11)** |
| Addresses weight stigma | Education and/ or strategies to increase resilience to weight stigma, bullying, teasing | 4 (6) | 3 (2) |
|  | Addresses weight-focused communication skills (e.g. how to communicate with peers/ family about weight, how to address weight-related comments from peers/ family) | 5 (8) | 7 (5) |
|  | Education to support network persons on weight stigma/teasing | 1 (2) | 3 (2) |
|  | **Interventions with any strategy in this cluster** | **8 (13)** | **9 (7)** |
| Psychosocial health related monitoring | Self-monitoring of thoughts, feelings, mood (e.g. mood diary) | 7 (11) | 5 (4) |
|  | Review/feedback on self-monitoring (e.g. mood diary) | 5 (8) | 2 (1) |
|  | Encourages self-assessment of overall wellbeing (e.g. reflective practice) | 6 (9) | 4 (3) |
|  | **Interventions with any strategy in this cluster** | **10 (16)** | **8 (6)** |
| Behaviour change strategies related to psychosocial issues | Encourages psychosocial health related goals with/ without review | 12 (19) | 6 (4) |
|  | Increases skills to manage psychosocial health (e.g. stress management) | 25 (39) | 25 (18) |
|  | Inclusion of peer/ social support strategies | 11 (17) | 6 (4) |
|  | **Interventions with any strategy in this cluster** | **26 (41)** | **25 (18)** |

## **Supplementary file 5: Frequency of delivery features across intervention arms (k) of all eligible trials (sensitivity analyses)**

Sensitivity analyses consisted of all 58 eligible studies with 139 intervention arms (k). This included original coding of all studies in primary analyses as well as 26 additional studies that could not be verified.

| **Cluster** | **Components** | **Frequency in  *all eligible*** **intervention arms (%)**  **(sensitivity analyses, k=139)** |
| --- | --- | --- |
| Why - Theory | Weight maintenance | 10 (7) |
|  | Weight loss | 90 (65) |
|  | Weight loss and maintenance | 31 (22) |
| Psychological theory or framework underpinning intervention | Cognitive behavioural therapy (CBT) | 19 (14) |
|  | Enhanced Cognitive Behavioural Therapy (CBT-E) | 0 (0) |
|  | Acceptance and Commitment therapy (ACT) | 3 (2) |
|  | Dialectical Behaviour Therapy (DBT) | 0 (0) |
|  | Family-based therapy (FBT) | 0 (0) |
|  | Interpersonal Therapy (IPT) for binge eating | 0 (0) |
|  | Trauma-informed care | 0 (0) |
|  | Compassion focussed therapy | 0 (0) |
|  | Motivational interviewing | 7 (5) |
|  | Other General theory | 33 (24) |
| Target population/recipient of the intervention – AGE GROUP | Adolescent | 0 (0) |
|  | Adult | 139 (100) |
| Target population/recipient of the intervention – WEIGHT Category | Overweight | 99 (71) |
|  | Obesity | 135 (97) |
|  | Severe Obesity | 113 (81) |
| Target population/recipient of the intervention – Individual vs with support person/s | Individual | 138 (99) |
|  | Individual with support person | 1 (1) |
|  | Family or household-based treatment approach | 0 (0) |
| What – materials | Information sheets/booklets | 75 (54) |
|  | Food | 9 (6) |
|  | Diet monitoring materials | 62 (45) |
|  | Supplements | 3 (2) |
|  | Meal replacement products | 9 (6) |
|  | Sports equipment | 2 (1) |
|  | Fitness/activity tracker | 34 (24) |
|  | Body scales | 16 (12) |
|  | Food scales | 3 (2) |
|  | Mobile app | 6 (4) |
|  | Website access (online resources) | 25 (18) |
|  | Social media | 3 (2) |
|  | Other materials | 30 (22) |
| What – procedures | Nutrition education | 82 (59) |
|  | Energy prescription or target | 64 (46) |
|  | Physical activity education | 60 (43) |
|  | Exercise classes | 27 (19) |
|  | Psychological component | 87 (63) |
| What – outcome measures | Weight/adiposity | 139 (100) |
|  | Physical health outcomes | 64 (46) |
|  | Psychosocial/Mental health outcomes | 93 (67) |
|  | Eating behaviour outcomes | 81 (58) |
|  | Individual weighing at visits | 114 (82) |
|  | Blind weighing at visits | 5 (4) |
|  | Group weighing | 0 (0) |
|  | Communication about the ability to decline weight or opt out of weighing during visits | 7 (5) |
| Who provided – intervention delivered by (personnel, training, qualifications) | Dietitian/nutritionist | 77 (55) |
|  | Nurse | 6 (4) |
|  | Exercise Physiologist/Physiotherapist/Personal trainer/Other exercise professional | 24 (17) |
|  | Psychologist/counsellor | 51 (37) |
|  | Physician – paediatrician, GP, endocrinologist | 17 (12) |
|  | Pharmacist | 0 (0) |
|  | Researcher/non-health professional | 24 (17) |
|  | Self-delivered | 12 (9) |
|  | Other | 20 (14) |
| Training for interventionist | Training for the interventionist | 40 (29) |
| How – delivery mode | Face-to-face | 127 (91) |
|  | Computer/Web-based/Online | 31 (22) |
|  | Call (telephone, video)/SMS | 40 (29) |
|  | Printed material | 57 (41) |
|  | Individual | 73 (53) |
|  | Group | 85 (61) |
|  | Peer support | 1 (1) |
| Where - Intervention setting | Hospital outpatient | 11 (8) |
|  | Hospital inpatient | 2 (1) |
|  | University/research centre | 42 (30) |
|  | Primary care | 7 (5) |
|  | Community | 3 (2) |
|  | School | 0 (0) |
|  | Household residence | 1 (1) |
|  | Virtual | 24 (17) |
|  | Commercial provider centres | 0 (0) |
|  | Workplace | 4 (3) |
| Post-intervention support | Referral to other services | 2 (1) |
|  | Additional information provided | 2 (1) |
|  | Other | 0 (0) |
| Tailoring | Was there an element of tailoring in the intervention? | 42 (30) |
| Modifications | Was the intervention modified? | 5 (4) |
| Fidelity | Were any intervention fidelity procedures (actual or planned) described? | 76 (55) |

**Delivery features – Intervention dose**

| **When and how much (intervention dose)** | **Median** | **Range** |
| --- | --- | --- |
| Total number of contacts | 24^a^ | 1-365 |
| Overall intervention duration (weeks) | 26^b^ | 4-104 |
| Total duration of contacts/sessions (minutes) | 1200^c^ (20 hours) | 40-4800 (0.67-80 hours) |

^a^18/139 (12.95%) could not be calculated

^b^10/139 (7.19%) could not be calculated

^c^65/139 (46.76%) could not be calculated

**Delivery features – Intervention intensity**

| **Intensity** | **Frequency in intervention arms (%)**  **k=139** |
| --- | --- |
| Less than weekly | 16 (11.5) |
| Weekly | 41 (29.5) |
| Every 2-3 weeks | 5 (3.6) |
| Monthly | 7 (5.0) |
| Greater than monthly | 2 (1.4) |
| Stage approach (e.g. weekly>monthly) | 58 (41.7) |
| Unable to calculate | 10 (7.2) |

References

1.	Lister N, Khalid R, Jardine I, Pryde S, Johnson B, Jebeile H. Delivery features and intervention strategies of weight management interventions that measure eating disorder outcomes in adolescents: a systematic review with intervention coding [Manuscript submitted for publication]. 2025.

2.	Lister NB, Jebeile H, Khalid R, Pryde S, Johnson BJ. Deconstructing complex interventions: piloting a framework of delivery features and intervention strategies for the eating disorders in weight-related therapy (EDIT) collaboration. Nutrients. 2023;15(6):1414.
